# Persistent enhancement of exciton diffusivity in CsPbBr_3_ nanocrystal solids

**DOI:** 10.1126/sciadv.adj2630

**Published:** 2024-02-21

**Authors:** Wenbi Shcherbakov-Wu, Seryio Saris, Thomas John Sheehan, Narumi Nagaya Wong, Eric R. Powers, Franziska Krieg, Maksym V. Kovalenko, Adam P. Willard, William A. Tisdale

**Affiliations:** ^1^ Department of Chemical Engineering, Massachusetts Institute of Technology, Cambridge, MA, USA.; ^2^ Department of Chemistry, Massachusetts Institute of Technology, Cambridge, MA, USA.; ^3^ Laboratory of Nanochemistry for Energy (LNCE), Institute of Chemical Sciences and Engineering (ISIC), École Polytechnique Fédérale de Lausanne, CH-1950 Sion, Switzerland.; ^4^ Department of Chemistry and Applied Bioscience, ETH Zürich, Zürich, Switzerland.; ^5^ Laboratory for Thin Films and Photovoltaics and Laboratory for Transport at Nanoscale Interfaces, Empa – Swiss Federal Laboratories for Materials Science and Technology, Dübendorf, Switzerland.

## Abstract

In semiconductors, exciton or charge carrier diffusivity is typically described as an inherent material property. Here, we show that the transport of excitons among CsPbBr_3_ perovskite nanocrystals (NCs) depends markedly on how recently those NCs were occupied by a previous exciton. Using transient photoluminescence microscopy, we observe a striking dependence of the apparent exciton diffusivity on excitation laser power that does not arise from nonlinear exciton-exciton interactions or thermal heating. We interpret our observations with a model in which excitons cause NCs to transition to a long-lived metastable configuration that markedly increases exciton transport. The exciton diffusivity observed here (>0.15 square centimeters per second) is considerably higher than that observed in other NC systems, revealing unusually strong excitonic coupling between NCs. The finding of a persistent enhancement in excitonic coupling may help explain other photophysical behaviors observed in CsPbBr_3_ NCs, such as superfluorescence, and inform the design of optoelectronic devices.

## Teaser

Perovskite nanocrystals exhibit an excitation memory effect that boosts excitonic coupling long after recombination.

## INTRODUCTION

In semiconducting materials, the diffusion of charge carriers or excitons—which are bound electron-hole pairs—is central to the operation of electrical devices, generating energy in the form of electricity, light, or heat. Recently, the development of time-resolved optical microscopy techniques has enabled direct visualization of electronic energy transport at the nanoscale (*1*–*11*). In particular, experimental access to nonequilibrium regimes of exciton/carrier transport has revealed previously unknown insights into mesoscale dynamics. At early times, anomalous regimes of superdiffusive and/or hot-carrier transport may be observed (*12*, *13*). At later times, the effect of energetic disorder manifests in subdiffusive transport phenomena, wherein the ensemble average diffusivity decreases over time (*14*). Here, we report the observation of a previously unidentified nonequilibrium transport modality in which the diffusivity of excitons in a nanocrystal (NC) array depends on how recently those NCs were previously in the excited state.

Perovskite materials have attracted much attention over the past decade due to their potential applications in optoelectronic devices, such as photovoltaic cells and light-emitting diodes (LEDs) (*15*–*22*). Direct visualization of charge carrier and exciton diffusion has been investigated in bulk (*6*, *23*–*26*) and two-dimensional (2D) (*27*–*29*) perovskites. CsPbBr_3_ NCs are a particularly attractive perovskite morphology due to their bright and stable luminescence (*15*, *30*), quantum optical properties (*31*–*33*), and evidence for strong excitonic coupling in NC arrays (*34*–*36*). Initial reports suggest highly mobile excitons within CsPbBr_3_ NC solids (*7*, *37*, *38*).

## RESULTS

### Imaging exciton transport in CsPbBr_3_ NC solids

**Fig. 1.**
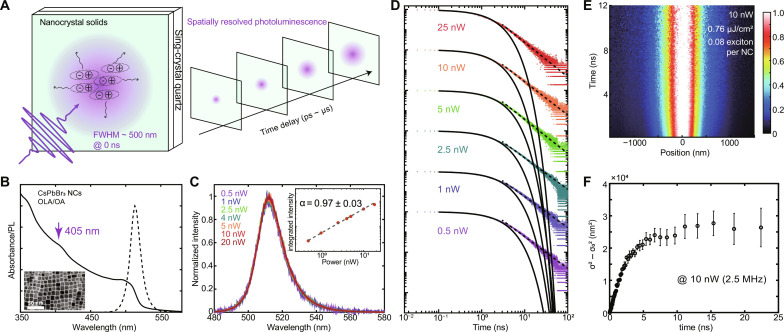
Transient photoluminescence microscopy (TPLM) of CsPbBr_3_ NC solids. (**A**) Schematic showing a near-diffraction-limited laser pulse generating a population of excitons, which subsequently diffuse within the film, leading to spatial broadening of the photoluminescence signal over time (laser excitation spot not to scale). (**B**) Normalized absorbance (solid line) and photoluminescence (dashed line) spectra of the CsPbBr_3_ (OLA-OA) NCs in toluene. The arrow indicates the laser excitation wavelength for all of the TPLM measurements in this study. The inset shows a transmission electron micrograph (TEM) image of the CsPbBr_3_ NC sample. (**C**) Normalized power-dependent emission spectra showing no peak shift as a function of excitation laser power (0.5 to 20 nW, 2.5 MHz, 500-nm-diameter laser spot). The inset shows the integrated photoluminescence intensity as a function of laser excitation power. The dashed line is a linear fit of the data with the fitted parameters labeled above. (**D**) Power-dependent transient photoluminescence plotted on a log-log scale (5-MHz repetition rate). The solid lines are single-exponential fits to the first 3 ns, and dashed lines are power-law fits to 3 to 20 ns. (**E**) Sample normalized TPLM color map plotted as a function of spatial position (*x* axis) and time (*y* axis) at 10 nW (2.5-MHz repetition rate). (**F**) Mean square displacement as a function of time extracted from the data shown in (E). See tables S1 and S2 for detailed measurement parameters.

To characterize exciton dynamics in CsPbBr_3_ NC solids, we used transient photoluminescence microscopy (TPLM) to track the radiative recombination of photogenerated excitons with both temporal and spatial resolution, as illustrated in Fig. 1A and fig. S1. Briefly, a variable repetition rate pulsed laser [405 nm, ~50 ps, full width at half maximum (FWHM) of 500 nm, 0.5 to 40 MHz] generated an initial population of excitons in a near-diffraction-limited spot. Epifluorescence was collected by a microscope objective lens and magnified by ~500× using a telescope, then an avalanche photodiode (APD; 50 μm by 50 μm active area) was raster scanned across the magnified imaging plane. Transient photoluminescence (PL) data were collected at each spatial position, allowing the time-dependent spatial distribution to be reconstructed and analyzed. The overall temporal resolution (~80 ps) is limited by the excitation laser pulse width and the APD response time. As a superresolution optical technique, the spatial resolution is ultimately limited by the total photon counts and other ancillary factors affecting the signal-to-noise ratio of the measurement, meaning that exciton diffusion lengths much smaller than the focused laser beam waist can be reliably determined for bright emitters (*2*). Further experimental details are listed under Materials and Methods, and a diagram of the instrument can be found in fig. S1.

We first investigated CsPbBr_3_ NCs capped with a mixture of oleylamine and oleic acid (OLA/OA) surface ligands, which were spun-cast into thin solid films supported on quartz glass substrates. The quasi-cubic CsPbBr_3_ NCs were 8.3 nm in size, and the solid film exhibited a PL quantum yield (QY) of 68%. The NC film absorption and emission spectra are shown in Fig. 1B. The transmission electron micrograph (TEM) image shows the quasi-cubic shape of the NCs (Fig. 1B, inset). The thin film samples were 30 to 40 nm in thickness and relatively uniform, as confirmed by atomic force microscopy (AFM) (fig. S2).

To ensure that TPLM experiments were conducted in the linear regime, we performed power-dependent PL spectroscopy to verify the absence of nonlinear multi-exciton interactions. Figure 1C shows normalized PL spectra collected with nominal laser power ranging from 0.5 to 20 nW at a constant repetition rate of 2.5 MHz focused to a ~500-nm-diameter laser spot, corresponding to 0.004 to 0.16 photons absorbed per NC per laser pulse—see table S1. Across this power regime, no peak shifting or broadening is observed; there is a linear relationship between integrated PL intensity and excitation laser power, indicating that additional non-radiative multi-exciton (i.e., Auger) processes are not introduced at higher power. The same linear trend was also observed when excitation laser power was increased under constant laser fluence and varying repetition rates (fig. S3).

As observed by others (*15*), the transient PL decay of CsPbBr_3_ NCs exhibits both prompt and delayed emission characteristics (Fig. 1D and fig. S4). The prompt emission, covering the first ~0 to 3 ns following photoexcitation, is well fit by a single-exponential decay curve (solid black lines on the log-log plot in Fig. 1D), while the delayed emission (≥3 ns) can be described by a power law (dashed line). While the origin of these complex emission dynamics is still debated, single-NC studies suggest that the prompt emission results from direct exciton recombination, whereas the delayed power-law emission arises from excitons that have undergone multiple trapping-detrapping processes (*39*). With increasing laser power, the prompt emission lifetime slightly decreased from ~2.3 to ~1.5 ns, while the power-law exponent for the delayed emission increased from ~1.5 to ~1.7 (fig. S4). Exciton dynamics in CsPbBr_3_ NCs are also affected by the exciton fine structure, consisting of multiple bright and dark sublevels. The bright-dark splitting in comparably-sized CsPbBr_3_ NCs is ~4 to 5 meV, and scattering among these levels is believed to be efficient at room temperature (*40*, *41*).

The results of one TPLM measurement are shown in Fig. 1E. These data were collected with a nominal laser power of 10 nW at a 2.5-MHz repetition rate and 500-nm laser spot size, corresponding to a laser pulse fluence of ~0.76 μJ/cm^2^, or 0.08 photogenerated excitons per NC per pulse (see note S1). To quantitatively extract exciton diffusivity from the TPLM data shown in Fig. 1E, the instantaneous spatial profile was fitted to a Gaussian shape at each delay time, and the mean square displacement (MSD), equal to the change in variance of the Gaussian distribution $\sigma {\left(t\right)}^{2}-\sigma_{0}^{2}$ , was extracted (see Materials and Methods and fig. S5) (*2*). The diffusivity of the exciton population is then extracted from the slope of the MSD curve. For normal diffusion, the MSD follows a linear relationship with time σ^2^(*t*) − σ^2^(*t* = 0) = 2*Dt*, where *D* is the diffusivity.

The MSD curve generated from the TPLM data in Fig. 1E is shown in Fig. 1F. The MSD grows linearly with time during the first ~3 ns and then becomes sublinear afterward, matching the transition from prompt to delayed emission in the transient PL data (Fig. 1D). The MSD behavior is consistent with the transient PL decay interpretation: At early times, excitons diffuse freely within the NC solid while spontaneously undergoing radiative recombination; at later times, exciton trapping begins to dominate the spatiotemporal dynamics, leading to a dramatic decrease in exciton diffusivity. For the remainder of this study, we focus exclusively on the early-time dynamics (<3 to 4 ns), corresponding to free exciton diffusion.

**Fig. 2.**
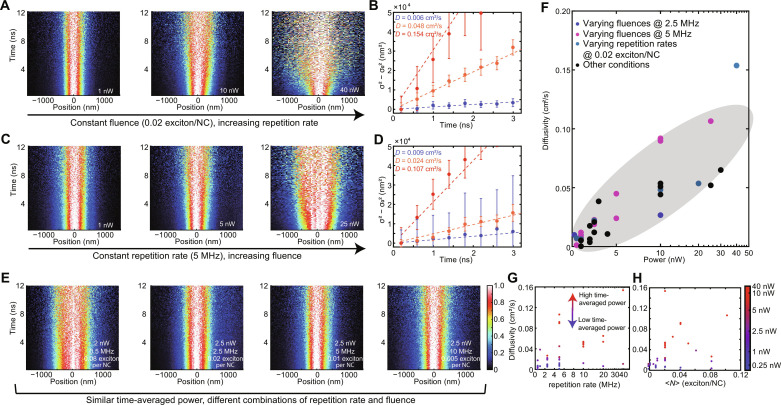
Dependence of measured exciton diffusivity on laser pulse fluence, repetition rate, and time-averaged power. (**A** and **B**) TPLM data with varying repetition rates while holding the pulse fluence constant at <*N* > = 0.02 absorbed photons per NC per laser pulse. (**C** and **D**) TPLM data with varying pulse fluence while holding the repetition rate constant (5 MHz). (**E**) TPLM data while varying pulse fluence and repetition rate together to achieve constant time-averaged power. (**F**) Exciton diffusivity measured under varying experimental conditions plotted versus time-averaged power. The gray oval is a guide to the eye. The *x* axis is in linear scale when power is less than 10 nW and logarithmic scale when power is above 10 nW. (**G** and **H**) Exciton diffusivity measured under varying experimental conditions plotted versus laser repetition rate or *<N*>*.* Data points are color-coded according to time-average power, indicated to the right.

TPLM experiments were performed while independently varying the laser repetition rate, laser pulse fluence (proportional to the excitation density, 〈*N*〉; see note S1), and, accordingly, the time-averaged laser power (Fig. 2). We observed that increasing either the fluence or repetition rate while holding the other variable constant always led to a greater measured value of the exciton diffusivity (Fig. 2, A to D). Unexpectedly, if the fluence and repetition rate were varied simultaneously such that the time-average power remained constant, then the measured value of the exciton diffusivity did not change (Fig. 2E and fig. S6). TPLM experiments performed under a variety of laser excitation conditions are aggregated and plotted together in Fig. 2 (F to H). While there is no correlation of diffusivity with either laser pulse fluence or laser repetition rate alone (Fig. 2, G and H), there is a strong positive correlation between diffusivity and time-averaged laser power (Fig. 2F).

### Exploring possible causes of power-dependent diffusivity

The observation of power-dependent diffusivity in the low excitation density regime is unexpected. Typically, exciton transport in semiconductor NC solids is understood in the framework of incoherent hopping mediated through dipole-dipole interactions, i.e., Förster resonant energy transfer (FRET) (*7*, *14*, *42*–*44*). In this picture, excitons act effectively as point dipoles that undergo discrete stochastic hopping transitions to neighboring NCs; varying the excitation laser power is expected to have no effect on the rate at which excitons move. We note that, at sufficiently high excitation density, 〈*N*〉, exciton-exciton interactions will introduce additional nonlinear recombination pathways that can interfere with the interpretation of time-resolved microscopy measurements (*2*, *3*); however, no dependence of the measured diffusivity on laser pulse fluence alone was observed (Fig. 2H). The correlation between exciton diffusivity and time-averaged laser power (Fig. 2F)—but not repetition rate or laser pulse fluence alone—cannot be explained within the traditional exciton random walk model. Consequently, we explored multiple experimental and material factors that might contribute to this anomalous observation, including (i) sample degradation, (ii) instrumentation/data analysis artifacts, (iii) laser heating, and (iv) surface effects.

**Fig. 3.**
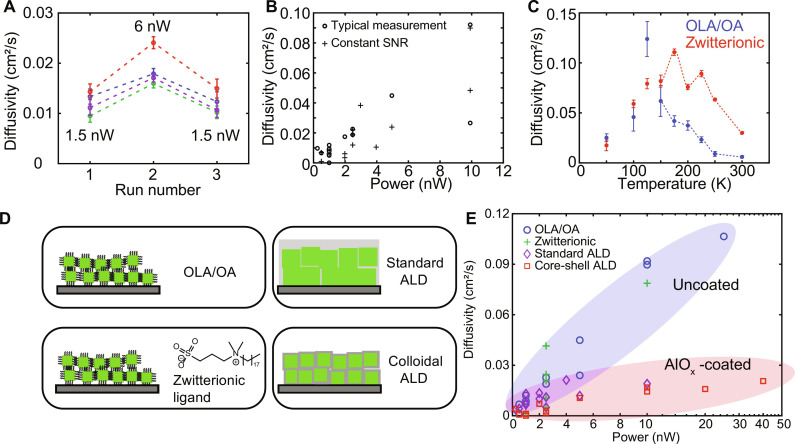
Measurement repeatability and effect of temperature, signal-to-noise ratio, and surface chemistry. (**A**) Demonstration that power-dependent diffusivity is a repeatable and reversible observation. A series of three consecutive TPLM experiments in which the excitation power was increased, and then decreased, was performed at four separate spots on the same sample. Each colored line comprising three data points corresponds to a unique location on the sample. (**B**) Invariance of the power-dependent trend with measurement signal-to-noise ratio. Open circles correspond to the typical TPLM measurement, in which the signal count rate varies naturally with the laser power used. Crosses correspond to a power series in which variable neutral density filters were placed in the signal path to achieve a constant signal-to-noise ratio across all powers. (**C**) Temperature-dependent exciton diffusivity of CsPbBr_3_ NCs capped with different ligands, as measured at 1 nW and 2.5 MHz. (**D**) Illustration of four different surface treatments investigated. Top left: The CsPbBr_3_ NCs capped with oleylamine and oleic acid (OLA/OA). Bottom left: The NC samples capped with zwitterionic ligands, 3-(*N*,*N*-dimethyloctadecylammonio)-propanesulfonate. Top right: The NC film coated with AlO_x_ using standard atomic layer deposition (ALD). Bottom right: The NC sample coated with AlOx using colloidal ALD growth. (**E**) Correlation of exciton diffusivity with time-averaged laser power for CsPbBr_3_ NC solids with different surface treatments. The *x* axis is in linear scale when power is less than 10 nW and in logarithmic scale when power is above 10 nW. The ovals are a guide to the eye, separately grouping the uncoated and AlO_x_-coated samples.

We first sought to verify that the power-dependent trend shown in Fig. 2F is repeatable and reversible. A series of three consecutive TPLM measurements were performed at four separate spots on a sample of CsPbBr_3_ NCs while non-monotonically varying the laser power (Fig. 3A). At each spot, we established a low-power baseline diffusivity by performing a measurement at 1.5 nW, then raised the power to 6 nW, then lowered the power back to 1.5 nW. At all four spots, the diffusivity at a power of 6 nW was higher than the diffusivity measured in the same location at a power of 1.5 nW. Moreover, at every spot, there was no statistically significant difference between the two diffusivities measured at powers of 1.5 nW in the first and third measurements, confirming that the low-power diffusivity value could be recovered after that same location was exposed to higher laser power.

Next, we investigated whether varying the signal-to-noise ratio of the TPLM measurement at different laser powers could lead to systematic errors in data analysis. In Fig. 3B, we compare a typical TPLM measurement series (open circles) to one in which variable neutral density filters were placed in the detection pathway to achieve a constant signal-to-noise ratio across all measurements (crosses). The two datasets overlap completely (albeit with some scatter in the data), demonstrating that differences in signal intensity or APD count rate are not responsible for the power-dependent trend. Moreover, we tested our data analysis procedure on simulated data with varying shot noise and background noise and found that diffusivities below 0.01 cm^2^/s could be reliably extracted from experimental data of the quality presented here (note S2).

### Effect of sample temperature and surface chemistry

Perhaps, the biggest concern in any power-dependent spectroscopy trend is the possible effects of laser heating. When a laser pulse is absorbed by the sample, heat is generated as the excess photon energy is dissipated over varying timescales. Typically, carrier-carrier scattering first leads to the formation of a Boltzmann distribution over the electronic degrees of freedom within ~100 fs (*45*), followed by hot carrier relaxation to the band edge via phonon emission on a picosecond timescale (*46*). As time-averaged power rises, more heat is generated, increasing the lattice temperature. The temperature rise is ultimately limited by thermal transport away from the laser excitation spot, a process whose timescale depends on the geometry of the measurement and the thermal conductivity of the sample. Thermal transport simulations of our TPLM experiment showed that, under typical laser fluence used here (~0.1 μJ/cm^2^), each absorbed laser pulse increases the local sample temperature by less than 0.01 K—with the majority of that heat dissipating within the first few nanoseconds following photoexcitation (note S3). These findings are consistent with experimental results obtained using time-resolved x-ray diffraction by Kirschner *et al.* (*47*), who found that excitation fluences on the order of a few millijoules per square centimeter (~10,000× larger than that used in our TPLM experiments) were required to increase the temperature of CsPbBr_3_ NCs by ~100 K and that the heat fully dissipated within ~10 ns.

To further investigate the potential consequences of sample heating, we directly measured the exciton diffusivity as a function of sample temperature (Fig. 3C). Temperature-dependent TPLM measurements were performed inside a closed-cycle liquid helium cryostat under vacuum (Montana Instruments, Cryostation). Two CsPbBr_3_ NC samples having similar size—but different surface chemistry—were investigated. One sample was terminated with a mixture of OLA/OA, while the other sample was terminated with a zwitterionic ligand, 3-(*N,N*-dimethyloctadecylammonio)propanesulfonate (*48*). In both samples, the diffusivity exhibited a similar non-monotonic trend— first increasing as the temperature decreased below room temperature, then eventually decreasing again as the sample was further cooled below ~150 K. We note that the diffusivity still had a positive correlation with the time-averaged laser power at lower temperatures (fig. S9). The full temperature-dependent behavior is the subject of ongoing investigation, but the salient observation here is the trend of decreasing diffusivity with increasing sample temperature near room temperature. If laser heating were responsible for the power-dependent trend shown in Figs. 2F and 3E, then we would expect the opposite behavior. Consequently, we conclude that laser heating is not responsible for the anomalous power-dependent trend in the data.

Unusual behavior in semiconductor NCs is sometimes associated with their unique surface properties. Next, we examined whether varying surface treatments led to the same power-dependent phenomenon. Four different types of samples were investigated: (1) the OLA/OA-capped NCs shown in Fig. 1, (2) NCs synthesized with a zwitterionic ligand, [3-(*N*,*N*-dimethyloctadecylammonio)-propanesulfonate], (3) spun-cast NC solids coated with AlO_x_ using standard atomic layer deposition (ALD) (*49*), and (4) NCs coated with an AlO_x_ shell grown via colloidal ALD before spin-casting (*50*), as illustrated in Fig. 3D. Samples 1, 3, and 4 were prepared at EPFL (Lausanne), while sample 2 was prepared at ETH (Zurich), before being shipped to MIT for transient microscopy measurements. The optical characterization of all samples is reported in figs. S10 and S11, and details of the sample preparation methods used are included in Materials and Methods.

In Fig. 3E, we compare the results of TPLM measurements made on the four samples with different surface treatments. All four samples show the same monotonic trend of increasing exciton diffusivity with increasing laser power. Similar to the OLA/OA samples shown in Fig. 2, there was no correlation between exciton diffusivity and laser pulse fluence or repetition rate alone (fig. S12). The repeated observation of power-dependent diffusivity in multiple CsPbBr_3_ NC samples synthesized by different laboratories and terminated with different chemistries suggests that this unexpected correlation is universal among CsPbBr_3_ NCs. The magnitude of the enhancement in diffusivity at higher laser powers was smaller in the samples with an AlO_x_ coating. However, it is difficult to separate the effects of these coatings on the intrinsic exciton diffusivity values observed at low powers (arising from, for instance, larger spacing between NCs) from the effects they may have on enhancing exciton diffusivity at higher powers. The sample with an AlO_x_ coating grown using ALD had similar exciton diffusivities compared to the uncoated samples at low powers but showed a smaller relative enhancement in exciton diffusivity at higher powers. On the other hand, the sample with a colloidally grown AlO_x_ coating exhibited lower absolute diffusivity than the uncoated NCs across all laser powers (fig. S13H). This is consistent with previous studies demonstrating slower exciton transfer rates between NCs with thicker surface shells (*14*). Moreover, when the colloidal AlO_x_ shell thickness increased from 4 layers to 8 or 12 layers, the diffusivity became too small to measure (fig. S13).

### Interpretation of power-dependent diffusivity

**Fig. 4.**
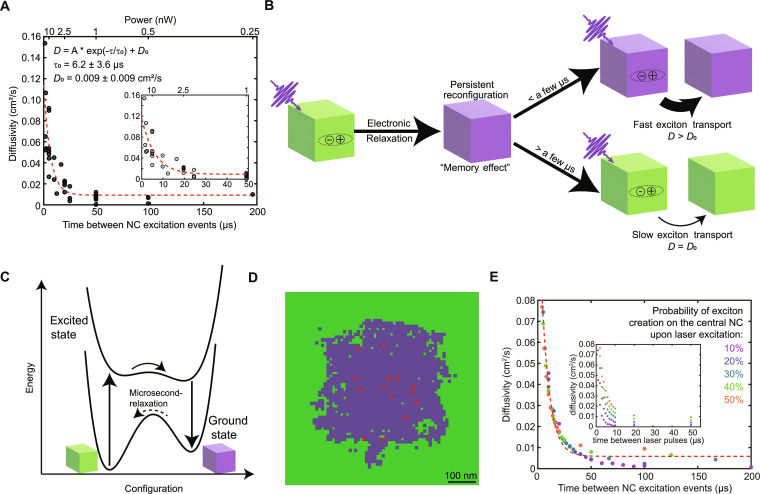
Persistent enhancement of exciton diffusivity. (**A**) Experimentally measured diffusivity relaxation curve. Exciton diffusivity of CsPbBr_3_ NCs with OLA/OA ligands as a function of time between NC excitation events. The mirror *x* axis shows the corresponding time-averaged power. The red dashed line is a single exponential fit to the data, and the best-fit parameters are annotated within the figure. The inset is a magnification of the early-time data points. (**B**) Schematic illustration of the excitation memory effect leading to persistent enhancement of exciton diffusivity. (**C**) Potential energy surface description of the phenomenon illustrated in (B). Black curves indicate the electronic excited state and electronic ground state of the NCs. (**D**) Snapshot of a kinetic Monte Carlo (KMC) simulation of exciton transport in a 2D NC array, which includes excitation memory effects. NCs in the relaxed state are shown in green and NCs in the metastable state are shown in purple. Red spots indicate the current location of excitons within the time-dependent simulation. (**E**) KMC simulation results plotted as a function of time between NC excitation events, showing consistency with the experimentally measured phenomenon. Different colors represent different simulated excitation fluences. The inset shows simulated diffusivity as a function of time between laser pulses (reciprocal of laser repetition rate). The red dashed line is a single exponential fit to the data.

To understand the observation of power-dependent diffusivity, we consider the microscopic meaning of time-averaged laser power in the context of this experiment. In the low excitation density regime (i.e., *<N >* less than 1), time-averaged power informs on the frequency of NC photoexcitation events; specifically, the inverse of time-averaged power is proportional to the average waiting time between NC excitation events. In Fig. 4A, we plot the measured exciton diffusivity versus time between NC excitations (proportional to 1/power) at room temperature. When plotted in this way, the data exhibit a characteristic exponential relaxation curve with an extracted relaxation time constant of ~6 μs. At 50 K, the exciton diffusivity follows a similar exponential relaxation curve when plotted versus the time between NC excitations, but with a substantially longer relaxation time constant of ~40 μs (fig. S14).

On the basis of our fitting parameters, the limiting diffusivity (as time between excitation events tends toward infinity) at room temperature is ~0.01 cm^2^/s, while the “enhanced” diffusivity measured under frequent photoexcitation conditions is up to 15× larger. The limiting diffusivity (~0.01 cm^2^/s) could also be recovered by lowering the laser repetition rate enough that the time between laser pulses was much longer than the relaxation time (~6 μs) inferred from high repetition rate measurements. Using sufficiently low repetition rates (16 or 32 μs between laser pulses), we were able to consistently measure diffusivities near the limiting value of 0.01 cm^2^/s (table S3).

The observation that exciton diffusivity changes as the exciton generation frequency on any given NC changes is unexpected. The relaxation time constant—the characteristic time it takes for a NC to relax to the low-diffusivity state—is on the microsecond timescale, which is two to four orders of magnitude longer than the exciton lifetime (Fig. 1). This striking observation suggests that CsPbBr_3_ NCs retain some persistent memory of previous exciton occupation events.

The exponential relaxation behavior shown in Fig. 4A and fig. S14 suggests that NCs can adopt two different states: a stable “slow state” associated with the low-diffusivity exciton transport regime and a metastable “fast state” that permits markedly increased exciton hopping rates. These states are illustrated schematically in Fig. 4 (B and C). To test our understanding, we built a phenomenological model of exciton transport that includes persistent excitation memory effects and studied model dynamics using kinetic Monte Carlo simulations (Fig. 4, D and E). In the model, the NC solid is represented as a 2D square lattice—each lattice site representing a single NC—with lattice spacing, *l* ≈ 10 nm, roughly corresponding to the nearest neighbor spacing.

In the model, NCs can adopt either a slow or fast state, depicted in Fig. 4D as green or purple squares, respectively. Excitons, depicted in red in Fig. 4D, occupy individual NCs and can hop between nearest neighbor NCs at a rate *k*_hop_ that depends on the state of the neighboring NCs. We find quantitative agreement between the model and experiment when excitons are assigned room-temperature hopping rates to slow- or fast-state NCs of $k_{\text{hop}}^{\left(\text{slow}\right)}=0.006\;{\text{ps}}^{-1}$ and $k_{\text{hop}}^{\left(\text{fast}\right)}=0.7\;{\text{ps}}^{-1}$ , respectively. The state of a NC (i.e., slow or fast) depends on the history of its exciton occupancy. Upon excitation—via laser pulse or hopping—a NC immediately transitions into the fast state and can remain in this state even if the exciton decays or hops away. We find agreement with experimental data when unoccupied fast-state NCs decay stochastically back to the slow state with a rate of *k*_relax_ = (8.1 μs)^−1^. Because of the difference between exciton (nanoseconds) and fast-state (microseconds) lifetimes, all NC relaxation is assumed to occur in the time between laser pulses, after all excitons have decayed. Figure 4D illustrates a snapshot of the simulation a few nanoseconds after one of the laser pulses. Figure 4E plots the simulated values of *D*_sim_ = [σ^2^(*t*) − σ^2^(0)]/2*t*, evaluated at *t* = 1 ns, for a range of simulations with differing pulse frequency and pulse intensity. We find that *D*_sim_ is primarily a function of the average time between NC photoexcitation as evaluated at the center of the Gaussian laser pulse, in near quantitative agreement with experimental observation.

## DISCUSSION

Exciton/charge carrier dynamics in NCs on microsecond timescales have often been discussed in the framework of photocharging, wherein an individual charge carrier (electron or hole) can remain on a NC for a few microseconds before full relaxation (*51*–*54*). Photocharging in CsPbBr_3_ NCs has been observed at higher laser pulse fluences at cryogenic temperatures (*39*) and is typically associated with electron (or hole) transfer to the NC surface. To investigate this possibility, we compared the diffusivity relaxation curves for OLA/OA-capped NCs (shown in Fig. 4A) to the colloidal AlO_x_-coated CsPbBr_3_ NCs (shown in fig. S15). Despite the presence of the insulating oxide layer on the AlO_x_-coated NC surface, the relaxation time constant was 10.8 μs—only slightly larger than the 6-μs value measured for the uncoated NCs. Moreover, photocharging is typically a nonlinear effect, requiring the interaction of one exciton with another exciton or photon, which we have shown is not the case in this study (Fig. 2H). Last, it is not clear why a charged NC should exhibit markedly enhanced excitonic coupling to its neighbors.

One hypothesis for the mechanism driving higher diffusivity measurements at higher laser powers is the presence of a distribution of long-lived trap states. In this model, excitons may become localized for variable lengths of time at trapping sites, preventing hopping to a neighboring NC. However, at higher laser powers, a greater proportion of these traps are likely to be filled, allowing excitons to move more freely within the NC film and increasing their apparent diffusivity. Such effects have been observed previously in transient microscopy experiments, including bulk and 2D perovskites (*10*, *28*, *55*). In this case, the metastable fast state depicted in Fig. 4 represents a NC whose trap state(s) are transiently filled, allowing subsequent excitons to pass more freely through that NC.

A more provocative explanation for the excitation memory effect illustrated in Fig. 4 (B and C) is the presence of a lattice polarization that persists long after exciton recombination. The existence of polarons—lattice deformations coupled to electronic excitations—is frequently invoked to explain experimental observations in halide perovskites (*56*–*61*). Particularly intriguing is the possibility of microscopic ferroelectric domains, which could act to collapse the oscillator strength along a particular NC axis. In this picture, the transition back to the relaxed/disordered state requires thermal energy from the surroundings, resulting in a long relaxation time that becomes even slower at lower temperatures, as shown in Fig. 4 (B and C) and fig. S14. Such behavior was recently reported in thin-film single-crystal CsPbBr_3_, where light illumination led to persistent enhancements in photoconductivity lasting on the order of 10^6^ s at low temperatures that were accompanied by structural changes, suggesting the presence of a relaxor-ferroelectric phase (*62*). However, the implications of these results on the structural dynamics and dipole strength of CsPbBr_3_ NCs are unclear, and the presence of ferroelectric-like behavior in halide perovskites is debated (*63*).

Regardless of the microscopic mechanism, the phenomenological observation of a persistent enhancement in excitonic coupling in CsPbBr_3_ NC solids is notable. In the low power limit, exciton diffusivity was measured to be around 0.01 cm^2^/s, which is already >10 times higher than the corresponding value in CdSe quantum dot solids (*64*–*68*). However, under the highest laser powers used in this study, the diffusivity reached 0.15 cm^2^/s [we note that Penzo *et al.* (*7*) reported a diffusivity of 0.5 cm^2^/s in similar CsPbBr_3_ NCs]. For reference, carrier diffusivity in bulk halide perovskite single crystals at room temperature has been estimated between 0.3 and 0.5 cm^2^/s, while diffusivity in perovskite thin films is typically an order of magnitude smaller, ~0.01 to 0.05 cm^2^/s (*25*, *26*, *69*, *70*). The observation of an exciton diffusivity in CsPbBr_3_ NC solids that exceeds the carrier diffusivity in perovskite thin films—despite the presence of long-chain organic ligands separating individual NCs—is striking.

Conventional FRET theory fails to fully explain exciton transport in CsPbBr_3_ NC arrays. The theoretical Förster radius for self-energy transfer in the OLA/OA-capped CsPbBr_3_ NCs is 9.5 ± 0.2 nm, whereas the measured diffusivity implies Förster radii ranging from 17.4 ± 0.8 nm to 25.6 ± 1.2 nm. This discrepancy corresponds to observed exciton transfer rates that are two to three orders of magnitude faster than their theoretical values (note S4). These unexpectedly large exciton diffusivity values indicate that CsPbBr_3_ NCs exhibit a particularly strong excitonic coupling that is further strengthened at higher excitation powers. Such behavior may be explained by partially coherent exciton transport, in which excitons hop between small domains of strongly coupled NCs (*71*), leading to an overall enhancement of the observed exciton diffusivity. There is growing evidence for strong dipole-dipole coupling in CsPbBr_3_ NC superlattices, as demonstrated through superfluorescence (*34*) and related phenomena (*36*). Last, the observation of power-dependent diffusivity has implications for the design of high-brightness LEDs (*21*, *72*–*79*) and lasers (*80*–*82*) featuring CsPbBr_3_ NCs.

## MATERIALS AND METHODS

### Material synthesis and characterization

#### CsPbBr_3_ NCs (OLA-OA) and CsPbBr_3_ NCs (AlO_x_) and their characterization at EPFL

##### Chemicals

The following reagents were used as received: cesium carbonate (Cs_2_CO_3_; 99.9%, Sigma-Aldrich), lead(II) bromide (PbBr_2_; 99.9%, Alfa Aesar), OLA (70% technical grade, Sigma-Aldrich), OA (90% technical grade, Sigma Aldrich), trimethylaluminum (TMA; 98%, Strem Chemicals), ethanol (EtOH; anhydrous, 95%, Sigma-Aldrich), methanol (95%, Sigma-Aldrich), 1-octadecene (ODE; 90% technical grade, Acros), octane (anhydrous, >99%, Sigma-Aldrich), hexane (anhydrous, >96%, TCI), and acetone (anhydrous extra dry, synthetic. grade, Acros Organics).

##### Perovskite nanocrystal synthesis

CsPbBr_3_ NCs were colloidally synthesized by a hot-injection method following previously reported and detailed procedures (*30*, *83*). The resulting NCs were washed in two steps: (i) centrifugation at 6000 rpm (~20 min) and redispersion in anhydrous hexane and (ii) addition of anhydrous acetone (0.5 volume ratio with hexane), centrifugation at 6000 rpm (~5 min) and redispersion in equal volumes of anhydrous hexane and octane, or just octane. Further dilutions were performed in accordance with the film preparation process.

##### Colloidal atomic layer deposition on perovskites NCs

Colloidal perovskite NCs were covered with amorphous aluminum oxide shells of different thicknesses following the previously reported and optimized c-ALD method developed by Loiudice *et al*. (*50*, *84*). An octane solution of perovskite NCs (typically with a NC concentration of ~8 mM) was placed in a three-necked flask connected to a standard N_2_ Schlenk line. The c-ALD surface treatment was then performed as follows: (1) the dropwise addition of 1 ml of TMA in octane (1.6 mM) to the reaction flask with a speed of 1 ml/hour, (2) a 5-min waiting time to ensure that the reaction in step 1 was completed, and (3) the addition of O_2_ gas by means of a mass flow controller. This three-step process is referred to as a single c-ALD cycle. The cycle is then repeated 4, 8, and 12 times to achieve increasing aluminum oxide shell thickness, which is then measured through dynamic light scattering.

##### Perovskite nanocrystal film preparation

CsPbBr_3_ NCs, both with and without c-ALD surface treatment, were deposited on 15 mm × 15 mm × 0.5 mm quartz substrates via spin-coating. All steps were performed in the glovebox under an inert N_2_ environment. Before spin-coating, the substrates were sonicated with consecutive cycles of acetone/isopropanol, and then treated with a toluene solution of (3-mercaptopropyl)trimethoxysilane (0.02 M) for 12 hours to improve NC adhesion to the surface. CsPbBr_3_ NC solutions with a hexane:octane (1:1 by volume) solvent ratio were spin-coated at 1000 rpm for 45 s to achieve optical densities of 0.01 to 0.07 at the first excitonic transition.

##### Atomic layer deposition

Low-temperature ALD was performed on a Savannah-200 ALD system from Cambridge Nanotech Inc., following the method from published work (*49*). Briefly, amorphous AlO_x_ was deposited on top of the CsPbBr_3_ NC films. TMA and ultrapure water were used as aluminum and oxygen sources, respectively. The reaction chamber was kept at a temperature of 50°C and an operating pressure of ~0.10 torr. The thickness of the deposited ALD layer was controlled by varying the number of ALD cycles, with 100 cycles equating to ~10-nm overcoating layer on top of the CsPbBr_3_ NC film.

##### Atomic force microscopy

AFM was used to measure the overall thickness and roughness of the perovskite composite films. The measurements were performed using a Nanoscope IIIa (Veeco, USA), operated in tapping mode, with Nanosensors PPP-NCSTR AFM probes. Thin lines were scratched on the samples to reveal the Si substrate. The mapping was carried out at the edge of the lines.

##### Transmission electron microscopy

TEM images were acquired on an Analytical JEOL-2100F FETEM equipped with a Gatan camera, using a beam energy of 120 kV. NC samples were dropcasted on Cu TEM grids (Ted Pella Inc.) before the imaging. Size measurements were performed using the software ImageJ and counting 200 particles per sample.

##### Steady-state absorption

Steady-state ultraviolet-visible (UV-vis) absorption measurements were performed in transmission mode using a PerkinElmer Lambda 950 spectrophotometer equipped with deuterium and tungsten halide lamps for UV and Vis-IR ranges, respectively. A photomultiplier tube (PMT) and Peltier-controlled PbS were used for detection.

##### Steady-state PL spectroscopy

Steady-state emission and QY PL measurements were recorded by a Horiba Jobin Yvon Fluorolog-3 spectrometer equipped with a PMT detector. All PL spectra were collected at an excitation wavelength of 370 nm. Absolute QY measurements were performed in a Spectralon-coated integrating sphere. For each sample, four measurements were performed: (i) sample emission (*S*_em_), (ii) blank glass emission (*B*_em_), (iii) sample excitation (*S*_exc_), and (iv) blank glass excitation (*B*_exc_). The absolute QY was then calculated as follows

$$\text{QY}=\frac{S_{\text{em}}-B_{\text{em}}}{B_{\text{exc}}-S_{\text{exc}}}$$ (1)

The reported QY values are the average of three measurements.

#### CsPbBr3 NCs (zwitterionic ligands) and their characterization at ETH

##### Chemicals

The following reagents were used as received: Cs_2_CO_3_ from Fluorochem; 3-(*N*,*N*-dimethyldodecylammonio)propanesulfonate (>99%, ASC12) from Roth; lead acetate trihydrate (99.99%), bromine (99.9%), ODE (technical grade), 3-(*N*,*N*-dimethyloctadecylammonio)propanesulfonate (>99%, ASC18), and OA (90%) from Sigma-Aldrich/Merck; toluene (for synthesis), acetone (HPLC grade), and ethylacetate (HPLC grade) from Thermo Fisher Scientific; and trioctylphosphine (TOP; >97%) from Strem.

##### Cs-oleate 0.4 M in ODE

Cs_2_CO_3_ (1.628 g, 5 mmol), OA (5 ml, 16 mmol), and ODE (20 ml) were evacuated at 25° to 120°C until the completion of gas evolution.

##### Pb-oleate 0.5 M in ODE

Lead(II) acetate trihydrate (4.607 g, 12 mmol), OA (7.6 ml, 24 mmol), and ODE (16.4 ml) were mixed in a three-necked flask and evacuated at 25° to 120°C until the complete evaporation of acetic acid and water.

##### TOP-Br2 0.5 M in toluene

TOP (6 ml, 13 mmol) and bromine (0.6 ml, 11.5 mmol) were mixed under an inert atmosphere. Once the reaction was complete and cooled to room temperature, the TOP-Br2 was dissolved in toluene (18.7 ml).

##### CsPbBr3 nanocrystals with ASC18 as a ligand

CsPbBr_3_ NCs were synthesized by dissolving Cs-oleate (4 ml, 1.6 mmol), Pb-oleate (5 ml, 2.5 mmol), and ASC18 (0.215 g, ca. 0.512 mmol) in ODE (5 ml) and heating the mixture under vacuum to 130°C, whereupon the atmosphere was changed to argon and TOP-Br_2_ in toluene (5 ml, 5 mmol of Br) was injected. The reaction was cooled immediately by an ice bath.

The crude solution (19 ml) was precipitated by the addition of acetone (10 ml) in a nitrogen-filled glovebox, followed by centrifugation at 29,500*g* (*g* is the Earth’s gravity) for 10 min. The precipitated fraction was dispersed in toluene (3 ml) and then washed three more times. Each time, the solution was mixed with two volumetric equivalents of acetone, centrifuged at 1300*g* for 10 min, and subsequently dispersed in progressively smaller amounts of the solvent (1.5 ml for the second cycle, 0.75 ml for the third cycle). After the last precipitation, NCs were dispersed in 1 ml of toluene and centrifuged at 1300*g* for 1 min to remove any non-dispersed residue.

##### Film formation

Films were spin-coated on single crystalline quartz substrates that were previously cleaned. Specifically, they were sonicated in soap water, cleaned with a water stream, and blow-dried. This sequence was repeated twice. The films were then sonicated in EtOH, blow-dried, sonicated in acetone, and blow-dried. The films were subsequently covered with a monolayer of hexamethyldisilazane (HMDS) and annealed in a nitrogen-filled glovebox. Thirty microliters of the solution were used per square centimeter of substrate, they were deposited, first spun at 500 rpm for 10 s followed by 1 min at 2000 rpm. For spin-coating, the NC solutions were diluted to inorganic NC mass (3 mg/ml).

##### Instrumentation

UV-vis absorption spectra of colloidal NCs were collected using Jasco V670 and Jasco V770 spectrometers in transmission mode. Fluorolog iHR320 Horiba Jobin Yvon was used to acquire steady-state PL spectra from solutions, using excitation at 350 nm. PL QYs of films and solutions were measured with Quantaurus-QY Absolute PL QY spectrometer from Hamamatsu. TEM images were collected using a Hitachi HT7700 microscope operated at 100 kV.

### Transient photoluminescence microscopy

NC solid samples were mounted on a piezo stage (attocube, ANC350) in a closed-cycle liquid helium cryostat under vacuum (Montana Instruments, Cryostation). For low-temperature measurements, the samples were cooled down to 5 K before being heated up with a temperature controller (Lakeshore, Model 335) to designated temperatures. Transient PL microscopy was performed using a home-built fluorescence microscope, as shown in fig. S1. NC solids were excited with a 405-nm pulsed laser (PDL 800-D, pulse width < 100 ps) at various repetition rates and fluences. The excitation laser pulses were spatially filtered by a single-mode optical fiber and focused down to a near-diffraction-limited spot by an objective lens mounted inside the vacuum chamber (Zeiss, EC Epiplan-Neofluar 100×/0.85 NA). Epifluorescence was collected by the same objective and filtered by a dichroic mirror (Semrock, Di02-R405) and a longpass colored glass filter (Thorlabs, FGL435M). The emission was then passed through a tube lens (Thorlabs, TTL200-S8) and a telescope (Thorlabs, AC254-030-A and AC254-125-A). The APD (Micro Photon Devices, timing resolution ~50 ps, active area 50 μm × 50 μm) was positioned at the imaging plane (×495 magnified) after the telescope. The position of the APD was controlled by two orthogonal motorized actuators (Thorlabs, ZFS25B). The evolution of the PL spatial profile with time was acquired by scanning the detector across the magnified emission profile and collecting a PL decay histogram at each position. For the samples in this study, a single dataset could be collected within 15 to 20 min.

Luminescence intensity was recorded as a function of time delay and spatial position. The general diffusion equation in one dimension is as follows

$$\frac{\partial n\left(x,t\right)}{\partial t}=D\left(t\right)\frac{\partial^{2}n\left(x,t\right)}{\partial x^{2}}-k\left(t\right)n\left(x,t\right)$$ (2)

where *n*(*x*, *t*) is the exciton density distribution as a function of time and location, *D*(*t*) is the time-dependent exciton diffusivity, and *k*(*t*) is the time-dependent exciton decay rate. For CsPbBr_3_ NCs, we assume that the diffusivity and decay rate are time-independent during the first 3 ns. In addition, we treat the laser pulse as an instantaneous source, $n\left(\tilde{x},0\right)$ . Then, the general solution to Eq. 3 could be written as

$$n\left(x,t\right)=e^{-\mathrm{kt}}\frac{1}{\sqrt{4\mathrm{\pi Dt}}}\int_{-\infty }^{\infty }n\left(\tilde{x},0\right)e^{-\frac{{\left(x-\tilde{x}\right)}^{2}}{4\mathrm{Dt}}}d\tilde{x}$$ (3)

As we normalize the PL emission at any given time delay, Eq. 4 can be rewritten into Eq. 5 as follows:

$$n\left(x,t\right)\propto \int_{-\infty }^{\infty }n\left(\tilde{x},0\right)\frac{1}{\sqrt{4\mathrm{\pi Dt}}}e^{-\frac{{\left(x-\tilde{x}\right)}^{2}}{4\mathrm{Dt}}}d\tilde{x}=n\left(\tilde{x},0\right)*G\left(x,t\right)$$ (4)

where $G\left(x,t\right)=\frac{1}{\sqrt{4\pi \mathrm{Dt}}}e^{-\frac{x^{2}}{4\mathrm{Dt}}}$ . Equation 5 shows that at any given time, the exciton density distribution is the convolution of the initial exciton density distribution created by the laser pulse and the Gaussian function. The variance of the Gaussian *G*(*x*, *t*) is σ^2^(*t*) = 2*Dt*. Therefore, by fitting the spatial profile at each time delay (fig. S2), the diffusivity could be extracted as

$$D=\frac{\sigma^{2}\left(t\right)-\sigma_{0}^{2}\left(t\right)}{2t}$$ (5)

where $\sigma^{2}\left(t\right)-\sigma_{0}^{2}\left(t\right)=\text{MSD}$ is the mean-squared displacement. In the MSD plots shown in the main text, the error bars on individual data points correspond to the SDs of the 25 neighboring data points. A line intercepting (0,0) was fitted to the data points between 0 and 3 ns, by which time more than 80% of the excitons have recombined. The error bars on diffusivity values correspond to the 95% confidence interval of the linear regression.

#### Steady-state PL spectroscopy

Steady-state PL spectroscopy was conducted with the same excitation conditions and sample mounting configuration as the TPLM measurements. After the tube lens, the emission is diverted using a silver mirror, collimated, refocused, coupled into a multimode fiber (Thorlabs, BFL 105LS02), and dispersed by a grating (300 gr/mm) inside a Princeton Instrument SP2150 spectrograph. Spectra were recorded using a thermoelectrically cooled charge-coupled device camera (Princeton Instrument Pixis 100).

### Theoretical model and kinetic Monte Carlo simulations

#### Description of the model

The NC solid was modeled as a square lattice with each lattice site representing an individual NC. Simulations were carried out on a 1000 × 1000 lattice to avoid any boundary effects. The lattice spacing was set to *l* = 10 nm. Each NC adopts one of two structural configurations: a slow configuration (the relaxed state of the lattice) and a fast configuration (the transient state admitting faster exciton transport). NCs in the fast state stochastically transition back to the slow state with a rate $k_{\text{relax}}=\tau_{\text{relax}}^{-1}$ , where τ_relax_ = 8.1 μs. Excitons occupy individual NCs and diffuse through nearest-neighbor hopping, as described below. Excitons decay stochastically with a rate $k_{\text{decay}}=\tau_{\text{ex}}^{-1}$ , where τ_ex_ = 2.5 ns is the exciton lifetime.

Excitons are created in pulses at increments of τ_pulse_, which is an adjustable parameter that sets the pulse frequency. At each increment, excitons are created at every lattice site in the system with Gaussian spatial distribution,

$$P_{\text{excite}}\left(x,y\right)=\alpha_{\text{pulse}}\text{exp}\left(-r_{\text{center}}^{2}/2\sigma_{\text{pulse}}^{2}\right)$$ (6)

where *x* and *y* denote the coordinates of a given lattice site (in units of *l*), α_pulse_ represents the intensity of the laser pulse ( $0<\alpha_{\text{pulse}}<1$ ), $r_{\text{center}}^{2}={\left(x-500\right)}^{2}+{\left(y-500\right)}^{2}$ , and σ_pulse_ = 10.62, to yield a full width at half maximum of 250 nm.

#### Model dynamics

Model dynamics were simulated with a simple Monte Carlo algorithm. Initially, there are no excitons and every NC is in the slow configuration. Whenever an exciton occupies a NC, via laser pulse or hopping, the NC immediately transitions into the mod configuration. Exciton hopping dynamics are then simulated with a 1-ps timestep until all excitons have decayed. At each timestep, each exciton attempts a hop to a randomly selected neighbor and hops with probability $P_{\text{hop}}^{\left(\text{fast}\right)}=0.7$ or $P_{\text{hop}}^{\left(\text{slow}\right)}=6\times 10^{-3}$ if the neighbor is in the fast or slow configuration, respectively. Following this, each exciton randomly decays (is eliminated from the simulation) with probability *P*_decay_ = 4 × 10^−4^ (selected to yield an exciton lifetime of 2.5 ns). Excitons are constrained to single occupancy, so if an exciton attempts to hop to an occupied site, its hopping probability is zero.

After all excitons have decayed, the NC configurations are relaxed over time increment to the next pulse. To do this, we select a random relaxation time, Δ*t*_relax_, for each NC in the mod configuration. Selecting Δ*t*_relax_ = −τ_relax_ ln (1 − *f*), where *f* is a random number between 0 and 1 yields a proper exponential distribution of configurational lifetimes. For a given NC, if Δ*t*_relax_ ≤ τ_pulse_, then the NC is returned to the ground state for the next simulated laser pulse. If Δ*t*_relax_ > τ_pulse_, then the NC remains in the mod state. Thus, as the pulse frequency nears τ_relax_, excitons can hop to NCs that transitioned into a mod state during the previous pulse.

For each set of α_pulse_ and τ_pulse_, we simulated a sequence of 1000 laser pulses, recording exciton positions every 50 timesteps. Time-dependent exciton density, ρ_ex_(*x*, *y*, *t*), was computed by averaging over the occupancy state of site (*x*, *y*) at time *t* after the most recent laser pulse for all 1000 pulses. We compute exciton diffusivity from the simulation in a manner analogous to the experiment. Specifically, we compute the mean radial exciton density as a function of time following the most recent laser pulse, ρ_ex_(*r*, *t*), assuming that the center of the laser pulse is located at the origin. We extract the time-dependent width of this distribution, σ^2^(*t*) = 〈*r*^2^(*t*)〉, where the angle brackets imply an average over ρ_ex_, and a simulated diffusivity as *D*_sim_ = [σ^2^(*t*) − σ^2^(0)]/2*t*, evaluated at *t* = 1 ns.

## Supplementary Materials

### This PDF file includes:

Notes S1 to S4 Figs. S1 to S15 Tables S1 to S3 References
