# Supplementary material for: Persistent enhancement of exciton diffusivity in CsPbBr3 nanocrystal solids
Source: Sci Adv. 2024 Feb 21;10(8):eadj2630. doi: 10.1126/sciadv.adj2630 (PMC10881049; doi:10.1126/sciadv.adj2630)
Supplement: Supplementary file 1 — Notes S1 to S4 Figs. S1 to S15 Tables S1 to S3 References [file sciadv.adj2630_sm.pdf]

Science Advances
AAAS

Supplementary Materials for

Persistent enhancement of exciton diffusivity in $CsPbBr_3$ nanocrystal solids

Wenbi Shcherbakov-Wu *et al.*

Corresponding author: William A. Tisdale, tisdale@mit.edu

Sci. Adv. **10**, eadj2630 (2024)
DOI: 10.1126/sciadv.adj2630

This PDF file includes:

Notes S1 to S4
Figs. S1 to S15
Tables S1 to S3
References

Supplementary Text

Supplementary Note 1: Calculation of Excitation Density

All TPLM measurements were conducted in a home-built microscope (see Materials and Methods). The focused laser spot on the sample surface is approximately Gaussian in spatial intensity profile with a 500 nm diameter (full width at half maximum). Nominal powers referenced here and in the main text were measured outside the microscope. The laser power throughput of the microscope was measured to be 37.4%, meaning that if nominal power is 1 nW, then the power at sample surface is

$$1\, nW * 37.4\% = 0.37\, nW.$$

The intensity of the laser power at the sample surface is then

$$\frac{0.37\, nW}{250nm^2 * \pi} = 0.19\, W/cm^2.$$

Reported values of laser intensity, fluence and excitation density correspond to the true value at sample surface. For a repetition rate of 2.5 MHz and nominal laser power of 1 nW, the laser pulse fluence is

$$\frac{0.19W/cm^2}{2.5*10^6\, s^{-1}} * \frac{\mu W}{10^{-6}W} = 0.076\mu J/cm^2.$$

To calculate excitation density the absorption coefficient, as well as the absorption cross section, must be determined first. For $CsPbBr_3$ NCs with an 8.3 nm edge length, the absorption coefficient at 400 nm (in toluene) was determined from the size-dependent molar extinction coefficient (*85*),

$$0.0242cm^{-1}\mu M^{-1}nm^{-3} * (8.3\, nm)^3 * \frac{10^6\mu M}{1M} = 1.38 * 10^7 cm^{-1}M^{-1}.$$

The absorption cross section of a single 8.3 nm $CsPbBr_3$ NC at 400 nm is then,

$$1.38 * 10^7 cm^{-1}M^{-1} * \frac{ln(10)*10^3 cm^3 L^{-1}}{6.02*10^{23} mol^{-1}} = 5.29 * 10^{-14} cm^2.$$

We assume that the absorption cross section at 405 nm is the same as this value, within error.

Then, the average number of photons absorbed per NC per laser pulse, <*N*>, (also referred to in the text as a unitless "excitation density") is,

$$\langle N \rangle = photons\ absorbed\ per\ NC\ per\ pulse$$

$$= absorption\ cross\ section\ [cm^2] * incident\ photons\ per\ laser\ pulse\ per\ unit\ area\ [cm^{-2}]$$

$$= 5.29 * 10^{-14} cm^2 * \frac{0.076(\mu J cm^{-2}) * \frac{1J}{10^6 \mu J}}{\left(\frac{1240eV * nm}{405\, nm}\right) * \frac{1.602 * 10^{-19} J}{1eV}}$$

$$= 0.0082$$

Supplementary Note 2: Simulation of TPLM Experimental Data

Because the exciton mean-squared displacement in TPLM experiments is comparable to the size of the focused laser beam, we turned to data simulation to understand limits of the data analysis procedures (**Fig. S7**). We assumed an initial spot size of 600 nm in FWHM, full-width half-max, ($\sigma(t=0) = \frac{600\ nm}{2*\sqrt{2*\ln(2)}}$) and an exciton diffusivity of 0.01 cm^2/s, similar to the experimental conditions. An experimentally obtained normalized lifetime trace $I(t)$ was added to simulate the data decay as a function of time. Therefore, the spatial profile at the laser arrival time could be expressed as:

$$n(x, t=0) = \frac{A * I(t=0)}{\sqrt{2\pi} * \sigma(t=0)} * e^{-\frac{x^2}{2\sigma(t=0)^2}} \quad \text{(S1)}$$

where A is a pre-factor modulating the intensity of the signal. Here, we arbitrarily set $A = 2 * 10^5$ to match the experimental signal intensity. At later times t, the variance and spatial distribution could be written as:

$$\sigma(t) = \sqrt{\sigma(t=0)^2 + 2Dt} \quad \text{(S2)}$$

$$n(x, t) = \frac{A * I(t)}{\sqrt{2\pi} * \sigma(t)} * e^{-\frac{x^2}{2\sigma(t)^2}} \quad \text{(S3)}$$

Both ambient background noise and shot noise were considered and added to the simulated PL emission data (**Fig. S7a**). Specifically, ambient background noise refers to both the dark current of the detector and the photons from the experiment surroundings that reached the detector. To recreate the noise, we created a matrix of uniformly distributed random numbers with the intensity similar to experimentally observed dark counts. In addition, shot noise describes the fluctuation of number of photons detected as photon detections are individual events. Shot noise is proportional to the square root of the experimental signal as:

$$I_{shot} \propto \sqrt{signal} \quad \text{(S4)}$$

For each data point, the shot noise is recreated as:

$$I_{shot} \propto rand(0,1) * \sqrt{signal} \quad \text{(S5)}$$

where $rand(0,1)$ is a random number generated between 0 and 1.

The same data analysis and fitting procedure were then applied to the simulated data set, and a diffusivity of 0.01 cm^2/s was recovered (**Fig. S7b-c**). It should be noted that only when the full spatial profile (when there were negligible PL counts towards both ends) was fitted and when the background counts were properly accounted for, could the diffusivity be accurately recovered; otherwise, a value much larger than 0.01 cm^2/s was extracted.

Supplementary Note 3: Heat Transport Simulation

To examine the effect of laser-induced heating on the nanocrystal temperature under experimental conditions, the temperature as a function of time and position in the nanocrystal was simulated. Using cylindrical coordinates, the time and spatial evolution of heat in the nanocrystal was modeled via the following partial differential equation:

$$\frac{\partial \Theta(r,z,t)}{\partial t} = D_{thermal} \nabla^2 \Theta(r,z,t) \tag{S6}$$

$\Theta(r,z,t)$ is the dimensionless temperature, where $\Theta = \frac{T(r,t) - T_{initial}}{\Delta T_{max}}$ as a function of radial position r, axial position z and time t. T is the actual temperature in the sample, $T_{initial}$ is the initial temperature that the sample is at before the laser excitation and ΔT_{max} is the maximum temperature rise induced in the sample as a result of the laser excitation. $D_{thermal}$ is the thermal diffusivity of the nanocrystal array; the representative value was obtained from Yang *et al* (*86*).

To calculate the most extreme scenario, it was assumed that all of the energy from the laser pulse was converted to heat energy instantaneously in the sample. Additionally, no-flux boundary conditions were used for all edges of the sample. The initial condition was derived from the measured experimental data by fitting a Gaussian shape to match the radial distribution of heat, and the axial heat distribution was approximated using Beer's law absorption at the excitation wavelength.

The partial differential equation was solved using an explicit finite differencing method. The results showed that the time required for the sample to reach less than 5% of the initial dimensionless temperature was on the same order of magnitude as the inverse of the repetition rate.

The maximum temperature rise induced in the sample as a result of the laser excitation ΔT_{max} was calculated using the experimental conditions:

$$\Delta E_{photon} \times \langle n \rangle = \rho_{bulk} c_{p,bulk} V_{NC\,bulk} \Delta T_{max} \tag{S7}$$

ΔE_{photon} is the photon energy, $\langle n \rangle$ is the average number of photons absorbed per pulse per nanocrystal, ρ_{bulk} is the bulk density of the nanocrystal, $c_{p,bulk}$ is the bulk specific heat capacity of the nanocrystal, $V_{NC\,bulk}$ is the bulk volume of the nanocrystal (using experimental dimensions). The resulting ΔT_{max} was found to be on the order of 0.01K, which is vanishingly small (**Fig. S8**). Despite the time required for the sample to return back to less than 5% of its initial dimensionless temperature being on the same order of magnitude as the inverse of the repetition rate, the effect of laser-induced heating on the temperature of the sample is expected to be negligible because the actual temperature rise induced in the sample is very low due to the low laser fluences used.

Supplementary Note 4: Förster Radius Calculations

Following Mork *et al* (*87*), the Förster radius R_0 in the OLA/OA-capped $CsPbBr_3$ nanocrystals was calculated using

$$R_0^6 = \frac{9\eta_{PL}\kappa^2}{128\pi^5 n^4} \int \lambda^4 F_D(\lambda)\sigma_A(\lambda)d\lambda\,. \tag{S8}$$

Here, η_{PL} is the quantum efficiency of the isolated NCs, assumed to be 0.7±0.1 based on the quantum yield of the nanocrystal film. κ^2 is a factor that accounts for the dipole orientation, assumed here to be 2/3, which is the value for random, freely rotating dipoles. n is the material's refractive index, here assumed to be equal to the volume-weighted average of the refractive indices of the $CsPbBr_3$ cores and the organic ligands. We use 2.7 as the refractive index of the $CsPbBr_3$ core (*88*), and 1.5 ± 0.1 as the refractive index of the ligands, a value representative of long hydrocarbon chains. We assume that the nanocrystals are perfectly cubic with an average interparticle spacing of 2.6 nm (*89*), giving an overall refractive index of n = 2.0 ± 0.1. $F_D(\lambda)$ is the normalized emission spectrum of the nanocrystals, and $\sigma_A(\lambda)$ is the absorption cross section of the nanocrystals. The spectrally dependent absorption cross section was calculated by scaling the measured absorption spectrum such that the absorption cross section at 400 nm was (5.29 ± 0.09) × 10^{-14} cm^2, as discussed in supplementary note 1 (*85*). We obtain a Förster radius of R_0 = 9.5 ± 0.2 nm. Since R_0 is raised to the sixth power in Eqn. (S8), it is robust to errors and uncertainties in the physical parameters. The overall uncertainty in R_0 almost entirely results from the uncertainty in the quantum yield.

Next, we calculate the FRET rate from the theoretical Förster radius. The FRET rate is related to the Förster radius by

$$k_{ET} = \frac{1}{\tau}\left(\frac{R_0}{d}\right)^6 \tag{S9}$$

k_{ET} is the FRET rate for a pair of nanocrystals, τ is the fluorescence lifetime of an individual nanocrystal, here estimated to be 2 ± 0.5 ns based on the initial fluorescence lifetimes of the nanocrystal films shown in Fig. S4. d is the distance between nanocrystals. We use d = 10.9 ± 0.4 nm based on the nanocrystal side length of 8.3 nm and assuming the OLA/OA ligands result in a surface-to-surface separation of 2.6 ± 0.4 nm (*89*). The theoretical Förster radius of 9.5 ± 0.2 nm corresponds to a per-pair FRET rate of k_{ET} = 0.2 ± 0.1 ns^{-1}.

We also calculated the Förster radius from our exciton diffusivity measurements. In a cubic lattice, the exciton diffusivity is related to the FRET rate by

$$D = k_{ET}d^2 \tag{S10}$$

where D is the diffusivity (*2*). Our diffusivity measurements ranged from about 0.01 cm^2/s at low excitation powers to about 0.1 cm^2/s at high excitation powers. Based on these estimates, our diffusivity measurements at lower excitation powers correspond to a pairwise FRET rate of k_{ET} = 8.4 ± 0.6 ns^{-1} and a Förster radius of R_0 = 17.4 ± 0.8 nm. The uncertainty in R_0 from the fluorescence lifetime alone is about 0.7 nm, and the uncertainty from the nanocrystal spacing alone is about 0.4 nm. Our diffusivity measurements at higher excitation powers correspond to a FRET

rate of k_{ET} = 84 ± 6 ns^{-1} and a Förster radius of R_0 = 25.6 ± 1.2 nm. The uncertainty in R_0 from the fluorescence lifetime alone is about 1.1 nm, and the uncertainty from the nanocrystal spacing alone is about 0.6 nm.

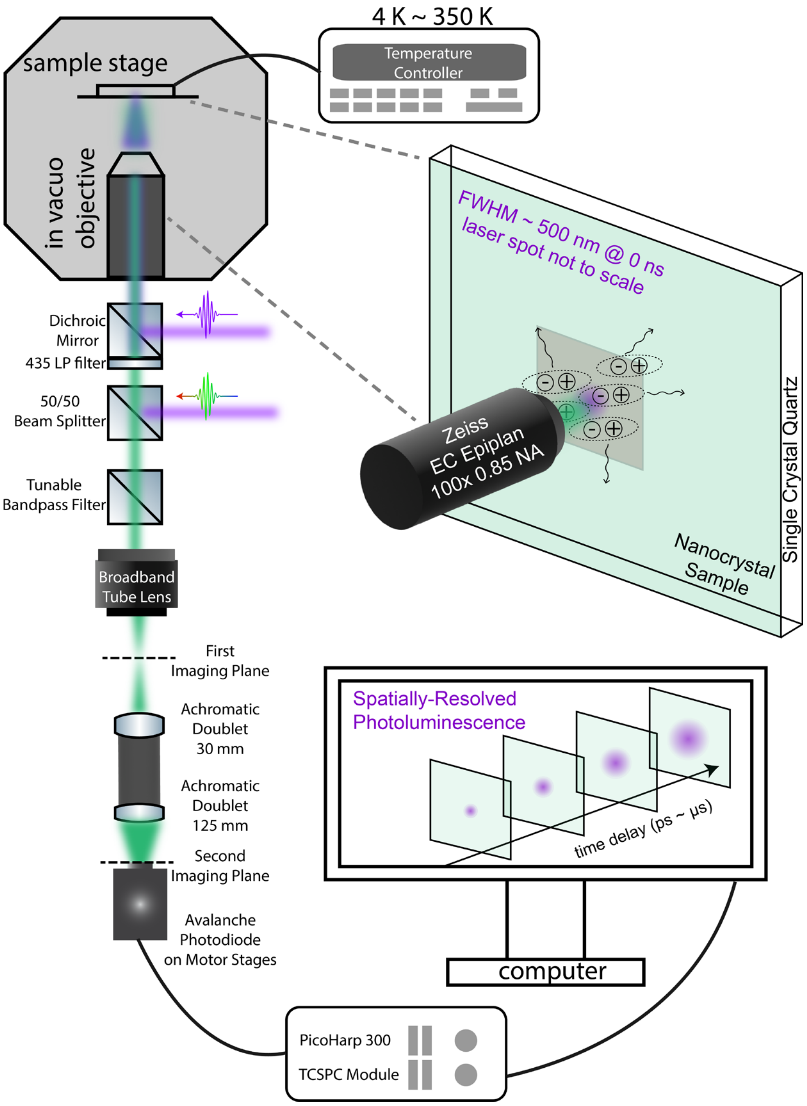

Fig. S1. Schematic of the home-built cryo-microscope used for TPLM.

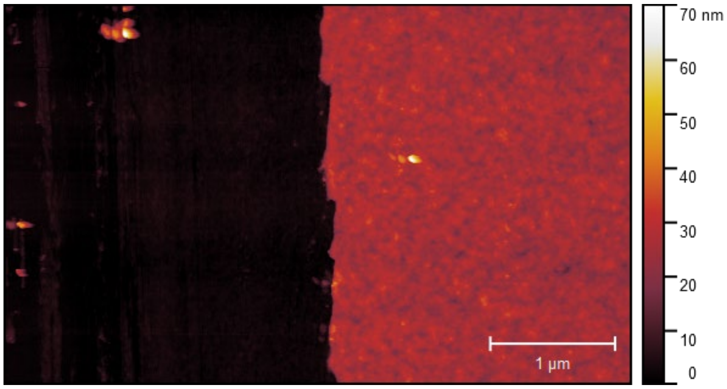

Fig. S2. Atomic Force Microscopy (AFM) image of sample NC solids (OLA-OA). The NC film has a uniform thickness of 30~40 nm.

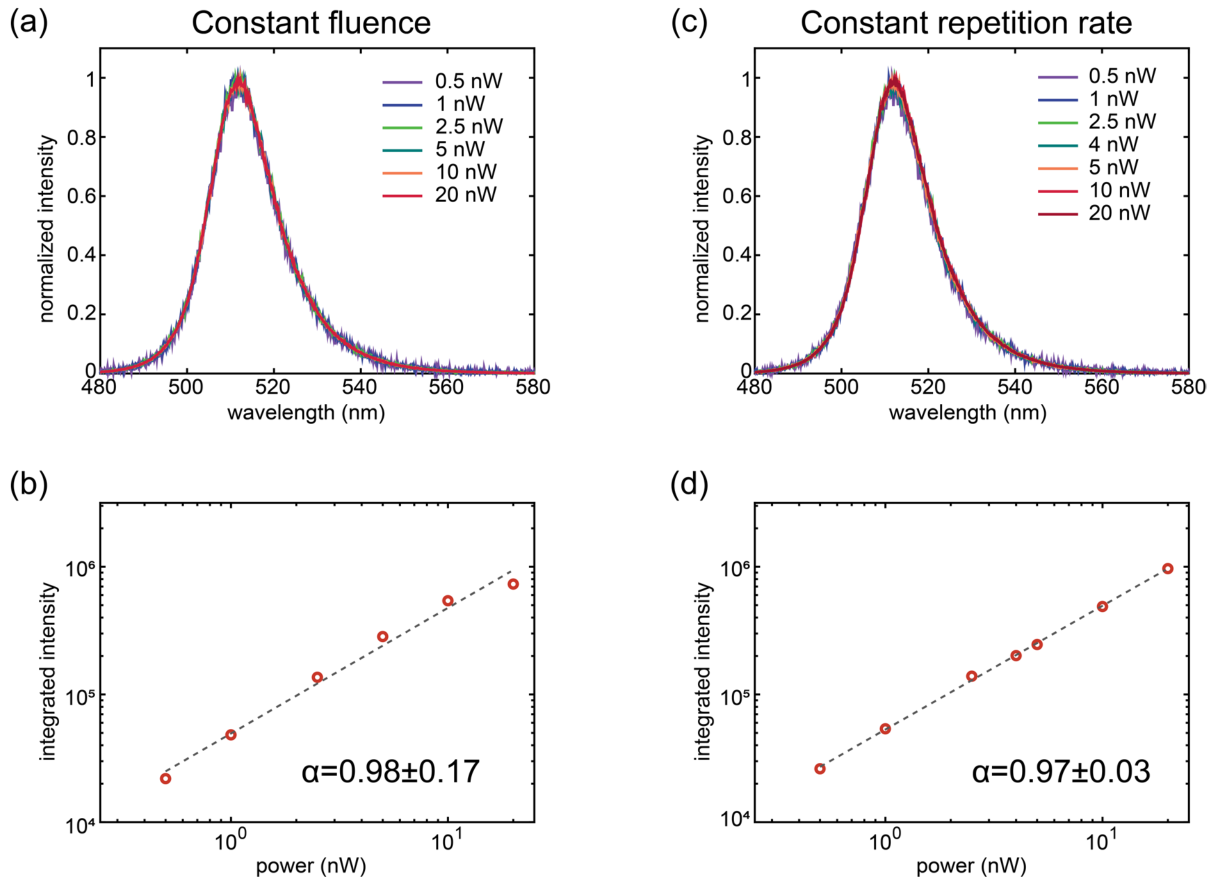

Fig. S3. Steady-state photoluminescence spectra of the $CsPbBr_3$ NC solid film at various power conditions. (a) Normalized PL spectra under constant fluence but various repetition rates. (b) Integrated intensity as a function of power (constant fluence, various repetition rates). The dash line is a linear fit to the data points with the exponent shown in the panel. (c) Normalized PL spectra under constant repetition rate but various fluences. (d) Integrated intensity as a function of power (constant repetition rate, various fluences). The dash line is a linear fit to the data points with the exponent shown in the panel.

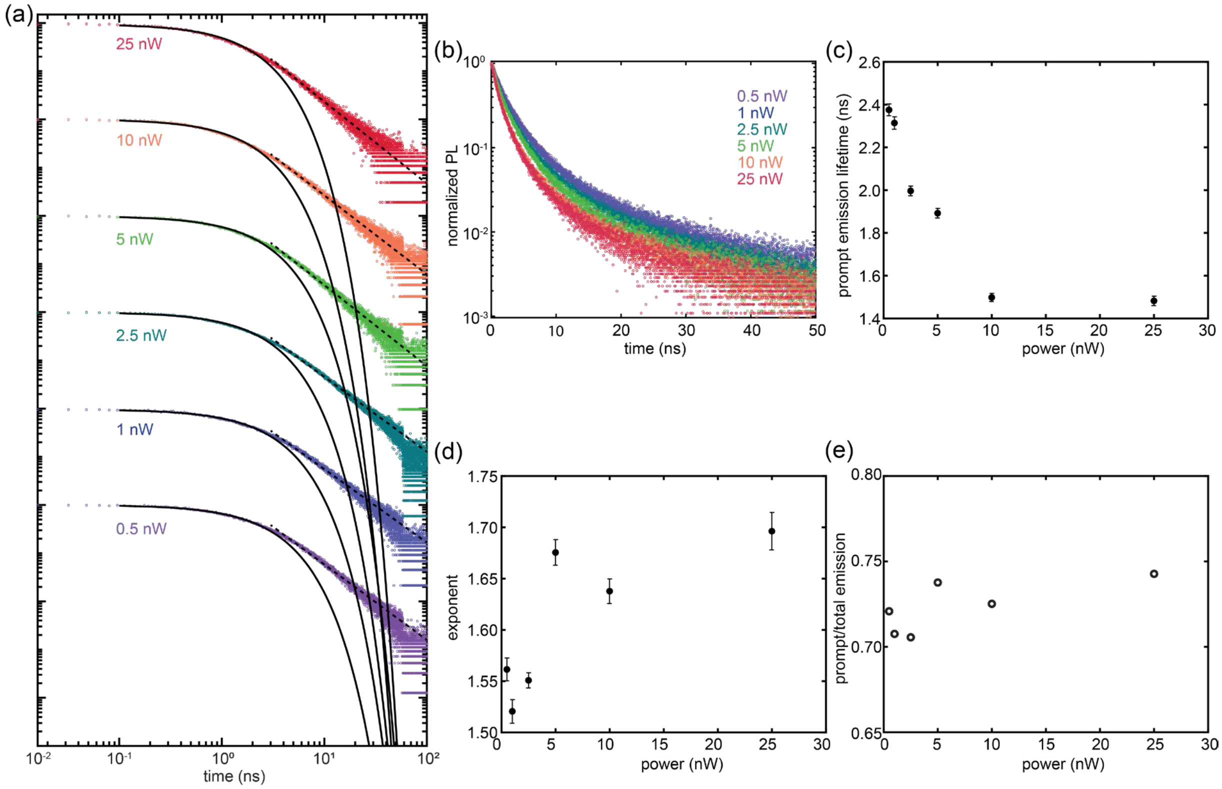

Fig. S4. Transient photoluminescence traces at various time-averaged power. (a-b) Power-dependent transient PL plotted on (a) log-log or (b) semi-log scale (all taken at 5 MHz). The solid lines are single exponential fits to the first 3 ns, and dash lines are power law fits to 3~20 ns. Same as Fig. 1d. (c-d) Extracted lifetime from the single exponential fit and extracted exponent from the power law fit for the two different time regimes. (e) The ratio between the prompt emission and total emission at various power.

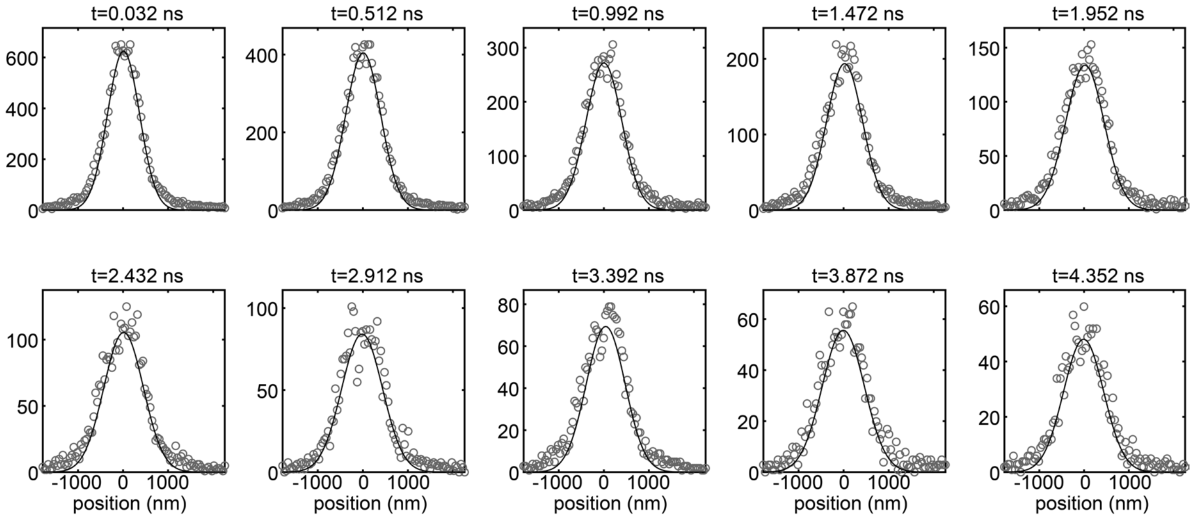

Fig. S5. Sample time slices of the TPLM measurements in $CsPbBr_3$ NC solids at 25 nW. Open circles are data points and solid lines are the fitted Gaussian shapes.

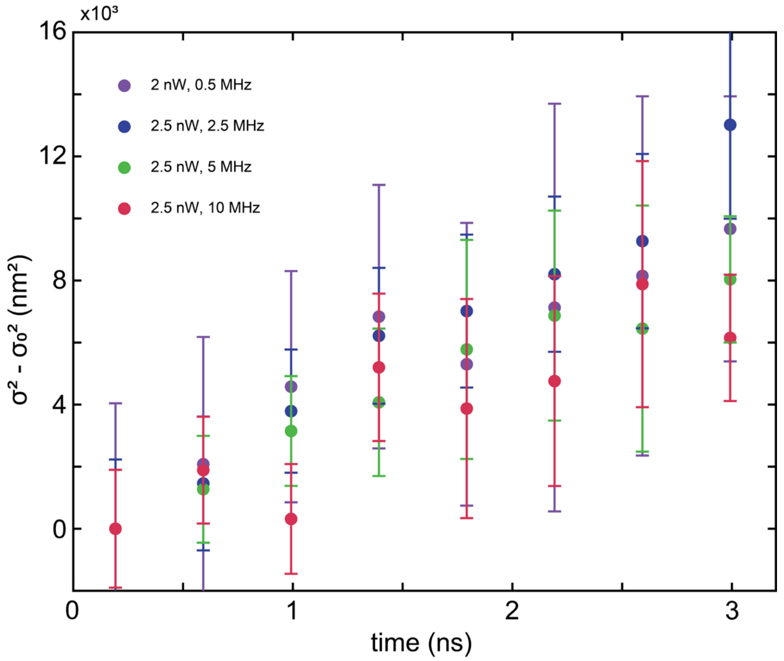

Fig. S6. Mean square displacement (MSD) curves while holding the time-averaged power constant and changing the repetition rates and fluences. The TPLM data are shown in **Fig. 2e**.

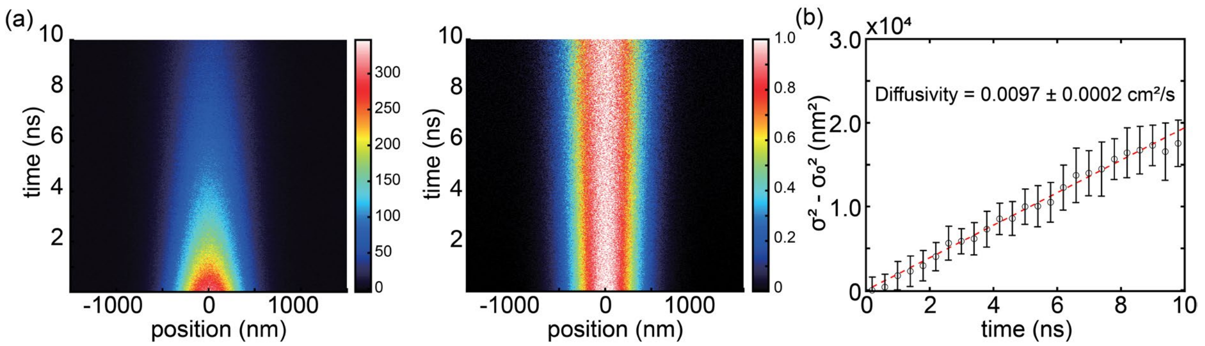

Fig. S7. Simulated data and analysis. (a) 2D color maps of unnormalized (left) and normalized (right) simulated transient photoluminescence microscopy data considering both dark counts and shot noise, assuming exciton diffusivity $D = 0.01\ cm^2/s$ and initial spot size (FWHM) of 600 nm. (b) Extracted mean square displacement as a function of time, recovering a diffusivity nearly identical to the simulation input.

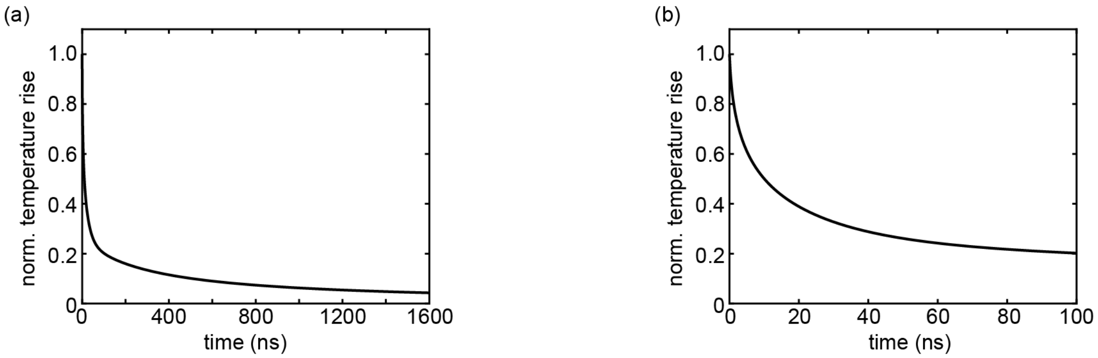

Fig. S8. Simulated heat transport as a result of laser heating on the sample. (b) shows the same data as panel (a), with a zoom-in view of the early-time dynamics.

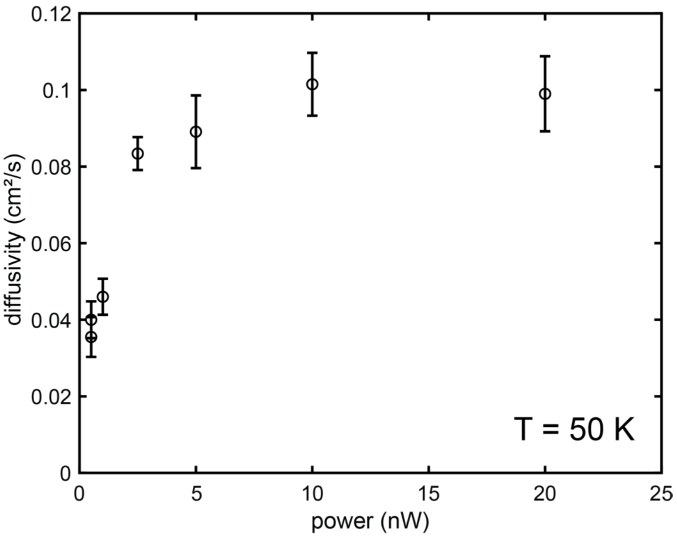

Fig. S9. Exciton diffusivity as a function of power at 50 K. Data taken on NCs terminated with the zwitterionic ligand 3-(*N,N*-dimethyloctadecylammonio)propanesulfonate.

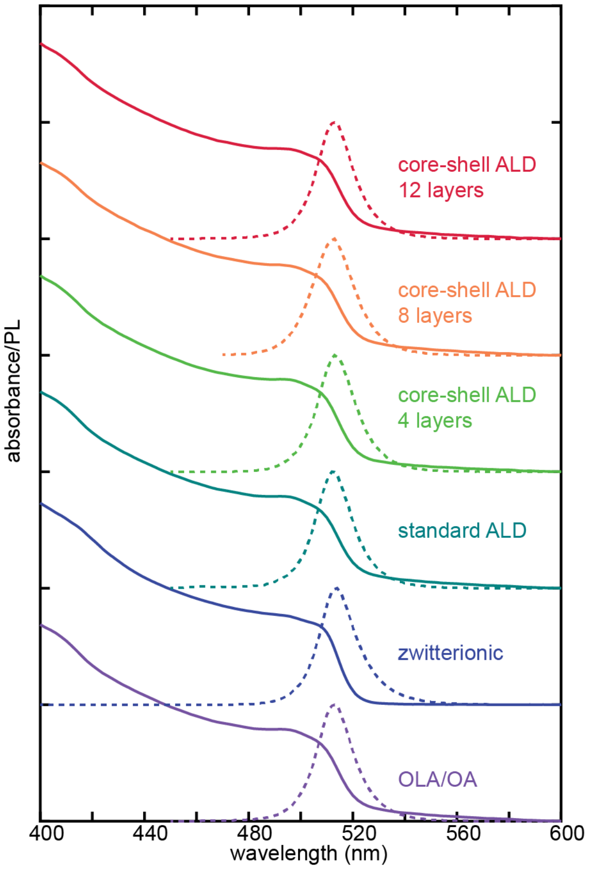

Fig. S10. Steady-state optical characterization of all the $CsPbBr_3$ NC solids studied. The solid lines correspond to absorbance and dash lines correspond to photoluminescence.

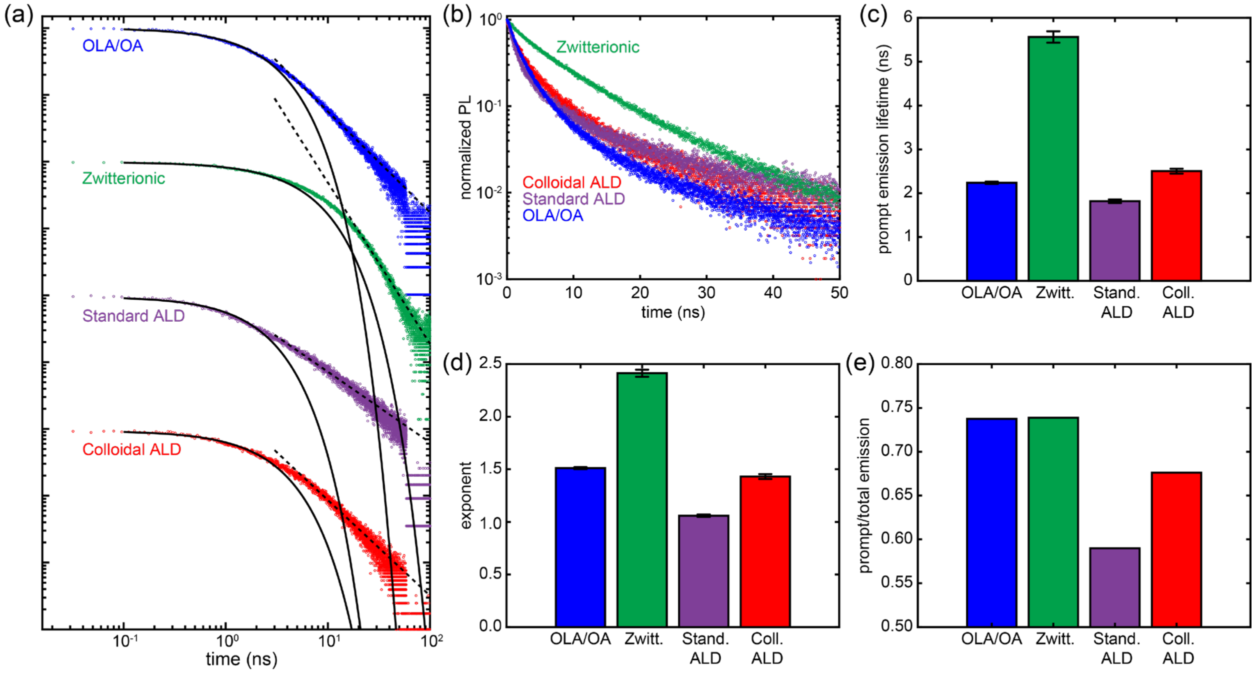

Fig. S11. Transient photoluminescence traces for samples with different surface treatments. (a-b) Transient PL at 1 nW plotted on (a) log-log or (b) semi-log scale. The solid lines are single exponential fits to the first 3 ns, and dash lines are power law fits to the parts of the PL decay at later times. (c-e) Extracted parameters for the PL decay traces for the different samples (Zwitt., Zwitterionic; Stand., Standard; Coll., Colloidal). (c) Lifetime from the single exponential fit at early times. (d) Exponent from the power law at later times. (e) The ratio between the prompt emission and total emission.

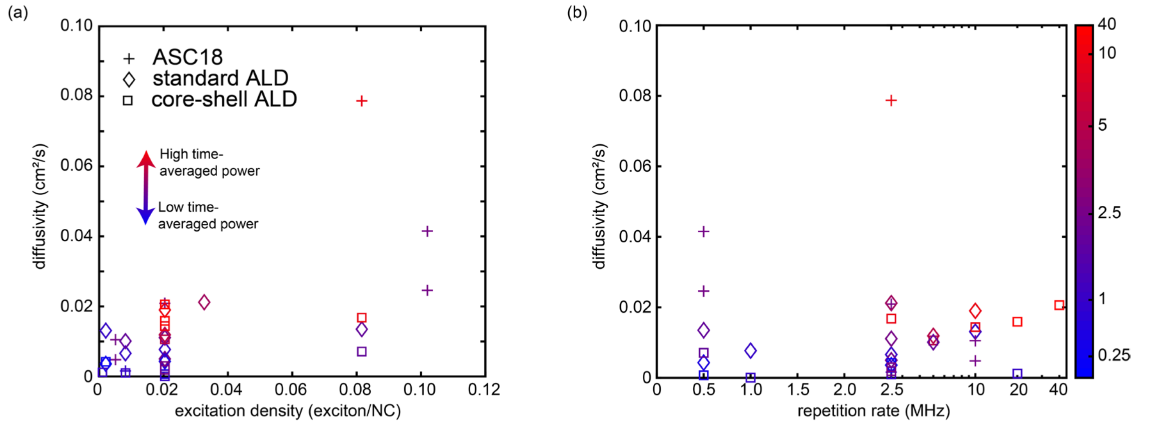

Fig. S12. Exciton diffusivity of samples with different surface treatments denoted with different markers. (a) Exciton diffusivity as a function of fluence. (b) Exciton diffusivity as a function of laser repetition rate. Data point colors in (a) and (b) correspond to the time-average power. The x-axis is in linear scale when repetition rate is less than 2.5 MHz and logarithm scale when repetition rate is above 2.5 MHz. Color map is shown on the right.

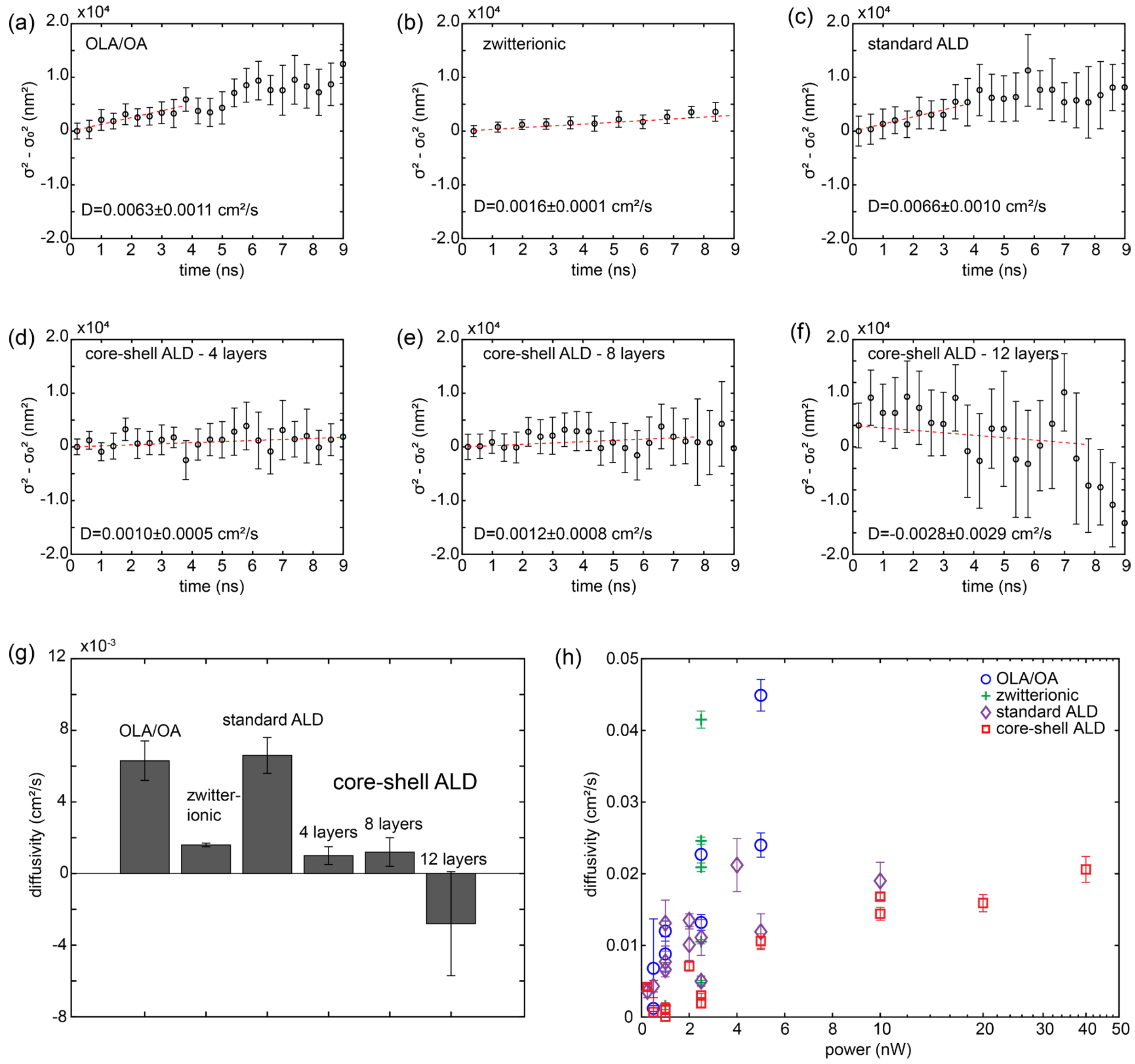

Fig. S13. Exciton transport measurements in $CsPbBr_3$ NC solid samples with varying surface treatments. (a-f) Mean square displacement as a function of time for all $CsPbBr_3$ NC solid samples under 1 nW; (a) OLA/OA capped $CsPbBr_3$ NCs. (b) Zwitterionic ligand (ASC18) capped $CsPbBr_3$ NCs. (c) $CsPbBr_3$ NCs with standard AlOx ALD layer. (d) $CsPbBr_3$ with colloidal AlOx ALD layers (4 layers). (e) $CsPbBr_3$ with colloidal AlOx ALD layers (8 layers). (f) $CsPbBr_3$ with colloidal AlOx ALD layers (12 layers). (g) Bar graph showing diffusivities of different samples under ~ 1 nW. (h) Zoomed in version of Fig. 3e with error bars to more clearly show differences among samples at low laser power.

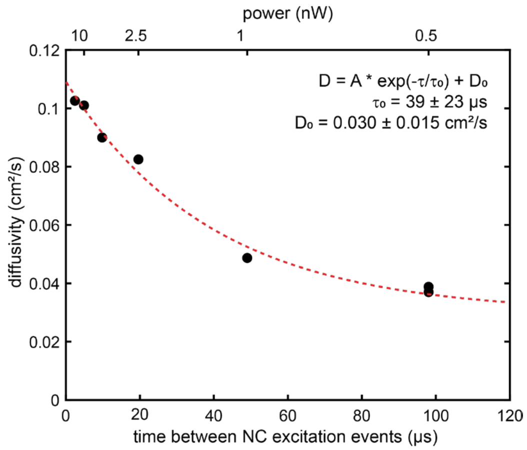

Fig. S14. Diffusivity relaxation curve at 50 K. Exciton diffusivity is plotted as a function of time between NC excitation events. Mirror *x*-axis indicates the time-averaged laser power. Diffusivities at each temperature are fit to a single exponential curve, with best fit parameters annotated within the figure.

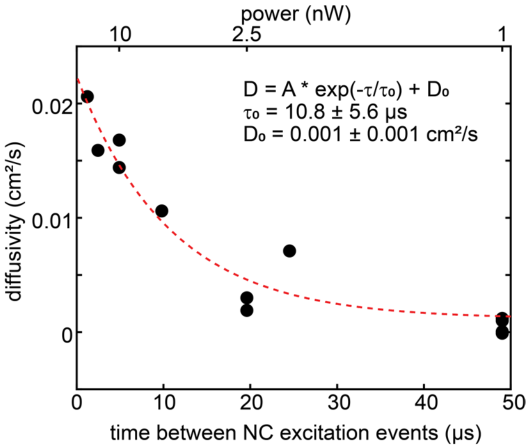

Fig. S15. Diffusivity relaxation curve of colloidal AlOx-coated $CsPbBr_3$ NCs. Exciton diffusivity of $CsPbBr_3$ NCs with 4 layers of colloidal AlOx ALD coating as a function of time between NC excitation events. Mirrored x-axis shows the corresponding time-averaged power. The red dash line is a single exponential decay fit. The fitted equation and parameters are indicated within the plot.

Table S1. Experimental conditions for the power-dependent photoluminescence spectra shown in Fig. 1c: excitation at 405 nm, 2.5 MHz, spot size FWHM ~500 nm.

Nominal power (nW)	Actual laser power @ sample surface (nW)	Intensity @ sample surface (W/cm^2)	Fluence @ sample surface ($\mu J/cm^2$)	$\langle N \rangle$ = Excitation density @ sample surface (exciton generated per NC per pulse)
0.5	0.185	0.095	0.038	0.0041
1	0.37	0.19	0.076	0.0082
2.5	0.925	0.475	0.19	0.0205
4	1.48	0.76	0.30	0.0326
5	1.85	0.95	0.38	0.041
10	3.7	1.9	0.76	0.082
20	7.4	3.8	1.52	0.163

Table S2. Experimental conditions for the transient photoluminescence data shown in Fig. 1d: excitation at 405 nm, 5 MHz, spot size FWHM ~500 nm.

Nominal power (nW)	Actual laser power @ sample surface (nW)	Intensity @ sample surface (W/cm^2)	Fluence @ sample surface ($\mu J/cm^2$)	$\langle N \rangle$ = Excitation density @ sample surface (exciton generated per NC per pulse)
0.5	0.185	0.095	0.019	0.00205
1	0.37	0.19	0.038	0.0041
2.5	0.925	0.475	0.095	0.01025
5	1.85	0.95	0.19	0.0205
10	3.7	1.9	0.38	0.041
25	9.25	4.75	0.95	0.1025

Table S3. Experimental conditions and exciton diffusivity values taken at low laser powers and low repetition rates. Data taken on OLA/OA-covered nanocrystals at room temperature. Uncertainties in exciton diffusivity values represent 95% confidence intervals.

Nominal power (nW)	Laser repetition rate (kHz)	Time between laser pulses (μs)	Pulse fluence @ sample surface (μJ/cm^2)	Exciton Diffusivity (cm^2/s)
0.4	31.25	32	2.4	0.011 ± 0.003
0.4	62.5	16	1.2	0.010 ± 0.002
0.9	62.5	16	2.7	0.011 ± 0.002

REFERENCES AND NOTES

1. G. M. Akselrod, P. B. Deotare, N. J. Thompson, J. Lee, W. A. Tisdale, M. A. Baldo, V. M. Menon, V. Bulovic, Visualization of exciton transport in ordered and disordered molecular solids. *Nat. Commun.* **5**, 3646 (2014).

2. N. S. Ginsberg, W. A. Tisdale, Spatially resolved photogenerated exciton and charge transport in emerging semiconductors. *Annu. Rev. Phys. Chem.* **71**, 1–30 (2020).

3. T. Zhu, J. M. Snaider, L. Yuan, L. Huang, Ultrafast dynamic microscopy of carrier and exciton transport. *Annu. Rev. Phys. Chem.* **70**, 219–244 (2019).

4. T. Zhu, Y. Wan, L. Huang, Direct imaging of frenkel exciton transport by ultrafast microscopy. *Acc. Chem. Res.* **50**, 1725–1733 (2017).

5. S. J. Yoon, Z. Guo, P. C. Dos Santos Claro, E. V. Shevchenko, L. Huang, Direct imaging of long-range exciton transport in quantum dot superlattices by ultrafast microscopy. *ACS Nano* **10**, 7208–15 (2016).

6. M. Delor, H. L. Weaver, Q. Yu, N. S. Ginsberg, Imaging material functionality through three-dimensional nanoscale tracking of energy flow. *Nat. Mater.* **19**, 56–62 (2020).

7. E. Penzo, A. Loiudice, E. S. Barnard, N. J. Borys, M. J. Jurow, M. Lorenzon, I. Rajzbaum, E. K. Wong, Y. Liu, A. M. Schwartzberg, S. Cabrini, S. Whitelam, R. Buonsanti, A. Weber-Bargioni, Long-range exciton diffusion in two-dimensional assemblies of cesium lead bromide perovskite nanocrystals. *ACS Nano* **14**, 6999–7007 (2020).

8. M. Liu, S. D. Verma, Z. Zhang, J. Sung, A. Rao, Nonequilibrium carrier transport in quantum dot heterostructures. *Nano Lett.* **21**, 8945–8951 (2021).

9. W. Li, M. S. R. Huang, S. K. Yadavalli, J. D. Lizarazo Ferro, Y. Zhou, A. Zaslavsky, N. P. Padture, R. Zia, Direct characterization of carrier diffusion in halide-perovskite thin films using transient photoluminescence imaging. *ACS Photonics* **6**, 2375–2380 (2019).

10. A. J. Goodman, D. H. Lien, G. H. Ahn, L. L. Spiegel, M. Amani, A. P. Willard, A. Javey, W. A. Tisdale, Substrate-dependent exciton diffusion and annihilation in chemically treated MoS2 and WS2. *J. Phys. Chem. C* **124**, 12175–12184 (2020).

11. M. Kulig, J. Zipfel, P. Nagler, S. Blanter, C. Schuller, T. Korn, N. Paradiso, M. M. Glazov, A. Chernikov, Exciton diffusion and halo effects in monolayer semiconductors. *Phys. Rev. Lett.* **120**, 207401 (2018).

12. Z. Guo, Y. Wan, M. Yang, J. Snaider, K. Zhu, L. Huang, Long-range hot-carrier transport in hybrid perovskites visualized by ultrafast microscopy. *Science* **356**, 59–62 (2017).

13. J. Sung, C. Schnedermann, L. Ni, A. Sadhanala, R. Y. S. Chen, C. Cho, L. Priest, J. M. Lim, H.-K. Kim, B. Monserrat, P. Kukura, A. Rao, Long-range ballistic propagation of carriers in methylammonium lead iodide perovskite thin films. *Nat. Phys.* **16**, 171–176 (2019).

14. G. M. Akselrod, F. Prins, L. V. Poulikakos, E. M. Y. Lee, M. C. Weidman, A. J. Mork, A. P. Willard, V. Bulović, W. A. Tisdale, Subdiffusive exciton transport in quantum dot solids. *Nano Lett.* **14**, 3556–3562 (2014).

15. A. Dey, J. Ye, A. De, E. Debroye, S. K. Ha, E. Bladt, A. S. Kshirsagar, Z. Wang, J. Yin, Y. Wang, L. N. Quan, F. Yan, M. Gao, X. Li, J. Shamsi, T. Debnath, M. Cao, M. A. Scheel, S. Kumar, J. A. Steele, M. Gerhard, L. Chouhan, K. Xu, X. G. Wu, Y. Li, Y. Zhang, A. Dutta, C. Han, I. Vincon, A. L. Rogach, A. Nag, A. Samanta, B. A. Korgel, C. J. Shih, D. R. Gamelin, D. H. Son, H. Zeng, H. Zhong, H. Sun, H. V. Demir, I. G. Scheblykin, I. Mora-Sero, J. K. Stolarczyk, J. Z. Zhang, J. Feldmann, J. Hofkens, J. M. Luther, J. Perez-Prieto, L. Li, L. Manna, M. I. Bodnarchuk, M. V. Kovalenko, M. B. J. Roeffaers, N. Pradhan, O. F. Mohammed, O. M. Bakr, P. Yang, P. Muller-Buschbaum, P. V. Kamat, Q. Bao, Q. Zhang, R. Krahne, R. E. Galian, S. D. Stranks, S. Bals, V. Biju, W. A. Tisdale, Y. Yan, R. L. Z. Hoye, L. Polavarapu, State of the art and prospects for halide perovskite nanocrystals. *ACS Nano* **15**, 10775–10981 (2021).

16. M. M. Lee, J. Teuscher, T. Miyasaka, T. N. Murakami, H. J. Snaith, Efficient hybrid solar cells based on meso-superstructured organometal halide perovskites. *Science* **338**, 643–647 (2012).

17. J. Jeong, M. Kim, J. Seo, H. Lu, P. Ahlawat, A. Mishra, Y. Yang, M. A. Hope, F. T. Eickemeyer, M. Kim, Y. J. Yoon, I. W. Choi, B. P. Darwich, S. J. Choi, Y. Jo, J. H. Lee, B. Walker, S. M. Zakeeruddin, L. Emsley, U. Rothlisberger, A. Hagfeldt, D. S. Kim, M. Gratzel, J. Y. Kim, Pseudo-halide anion engineering for α-FAPbI3 perovskite solar cells. *Nature* **592**, 381–385 (2021).

18. H. J. Snaith, Present status and future prospects of perovskite photovoltaics. *Nat. Mater.* **17**, 372–376 (2018).

19. H. Li, W. Zhang, Perovskite tandem solar cells: From fundamentals to commercial deployment. *Chem. Rev.* **120**, 9835–9950 (2020).

20. X. Zheng, Y. Hou, C. Bao, J. Yin, F. Yuan, Z. Huang, K. Song, J. Liu, J. Troughton, N. Gasparini, C. Zhou, Y. Lin, D.-J. Xue, B. Chen, A. K. Johnston, N. Wei, M. N. Hedhili, M. Wei, A. Y. Alsalloum, P. Maity, B. Turedi, C. Yang, D. Baran, T. D. Anthopoulos, Y. Han, Z.-H. Lu, O. F. Mohammed, F. Gao, E. H. Sargent, O. M. Bakr, Managing grains and interfaces via ligand anchoring enables 22.3%-efficiency inverted perovskite solar *cells Energy* **5**, 131–140 (2020).

21. X. K. Liu, W. Xu, S. Bai, Y. Jin, J. Wang, R. H. Friend, F. Gao, Metal halide perovskites for light-emitting diodes. *Nat. Mater.* **20**, 10–21 (2021).

22. K. Ji, M. Anaya, A. Abfalterer, S. D. Stranks, Halide perovskite light-emitting diode technologies. *Adv. Opt. Mater.* **9**, 2002128 (2021).

23. Z. Guo, J. S. Manser, Y. Wan, P. V. Kamat, L. Huang, Spatial and temporal imaging of long-range charge transport in perovskite thin films by ultrafast microscopy. *Nat. Commun.* **6**, 7471 (2015).

24. C. L. Kennedy, A. H. Hill, E. S. Massaro, E. M. Grumstrup, Ultrafast excited-state transport and decay dynamics in cesium lead mixed halide perovskites. *ACS Energy Lett.* **2**, 1501–1506 (2017).

25. A. Sridharan, N. K. Noel, H. Hwang, S. Hafezian, B. P. Rand, S. Kéna-Cohen, Time-resolved imaging of carrier transport in halide perovskite thin films and evidence for nondiffusive transport. *Phys. Rev. Materials* **3**, 125403 (2019).

26. D. W. deQuilettes, R. Brenes, M. Laitz, B. T. Motes, M. M. Glazov, V. Bulović, Impact of photon recycling, grain boundaries, and nonlinear recombination on energy transport in semiconductors. *ACS Photonics.* **9**, 110–122 (2021).

27. S. Deng, E. Shi, L. Yuan, L. Jin, L. Dou, L. Huang, Long-range exciton transport and slow annihilation in two-dimensional hybrid perovskites. *Nat. Commun.* **11**, 664 (2020).

28. M. Seitz, A. J. Magdaleno, N. Alcazar-Cano, M. Melendez, T. J. Lubbers, S. W. Walraven, S. Pakdel, E. Prada, R. Delgado-Buscalioni, F. Prins, Exciton diffusion in two-dimensional metal-halide perovskites. *Nat. Commun.* **11**, 2035 (2020).

29. J. D. Ziegler, J. Zipfel, B. Meisinger, M. Menahem, X. Zhu, T. Taniguchi, K. Watanabe, O. Yaffe, D. A. Egger, A. Chernikov, Fast and anomalous exciton diffusion in two-dimensional hybrid perovskites. *Nano Lett.* **20**, 6674–6681 (2020).

30. L. Protesescu, S. Yakunin, M. I. Bodnarchuk, F. Krieg, R. Caputo, C. H. Hendon, R. X. Yang, A. Walsh, M. V. Kovalenko, Nanocrystals of cesium lead halide perovskites (CsPbX3, X = Cl, Br, and I): Novel optoelectronic materials showing bright emission with wide color gamut. *Nano Lett.* **15**, 3692–6 (2015).

31. H. Utzat, W. Sun, A. E. K. Kaplan, F. Krieg, M. Ginterseder, B. Spokoyny, N. D. Klein, K. E. Shulenberger, C. F. Perkinson, M. V. Kovalenko, M. G. Bawendi, Coherent single-photon emission from colloidal lead halide perovskite quantum dots. *Science* **363**, 1068–1072 (2019).

32. G. Rainò, H. Utzat, M. G. Bawendi, M. V. Kovalenko, Superradiant emission from self-assembled light emitters: From molecules to quantum dots. *MRS Bull.* **45**, 841–848 (2020).

33. C. Zhu, M. Marczak, L. Feld, S. C. Boehme, C. Bernasconi, A. Moskalenko, I. Cherniukh, D. Dirin, M. I. Bodnarchuk, M. V. Kovalenko, G. Raino, Room-temperature, highly pure single-photon sources from all-inorganic lead halide perovskite quantum dots. *Nano Lett.* **22**, 3751–3760 (2022).

34. G. Rainò, M. A. Becker, M. I. Bodnarchuk, R. F. Mahrt, M. V. Kovalenko, T. Stöferle, Superfluorescence from lead halide perovskite quantum dot superlattices. *Nature* **563**, 671–675 (2018).

35. I. Cherniukh, G. Raino, T. Stoferle, M. Burian, A. Travesset, D. Naumenko, H. Amenitsch, R. Erni, R. F. Mahrt, M. I. Bodnarchuk, M. V. Kovalenko, Perovskite-type superlattices from lead halide perovskite nanocubes. *Nature* **593**, 535–542 (2021).

36. D. D. Blach, V. A. Lumsargis, D. E. Clark, C. Chuang, K. Wang, L. Dou, R. D. Schaller, J. Cao, C. W. Li, L. Huang, Superradiance and exciton delocalization in perovskite quantum dot superlattices. *Nano Lett.* **22**, 7811–7818 (2022).

37. M. Lorenzon, M. Jurow, M. J. Hong, Y.-H. Lu, E. S. Barnard, M. Salmeron, Y. Liu, E. Penzo, A. M. Schwartzberg, A. Weber-Bargioni, Improved stability and exciton diffusion of self-assembled 2D lattices of inorganic perovskite nanocrystals by atomic layer deposition. *Adv. Opt. Mater.* **8**, 2000900 (2020).

38. M. Yang, P. Moroz, E. Miller, D. Porotnikov, J. Cassidy, C. Ellison, X. Medvedeva, A. Klinkova, M. Zamkov, Energy transport in CsPbBr3 perovskite nanocrystal solids. *ACS Photonics* **7**, 154–164 (2019).

39. M. A. Becker, C. Bernasconi, M. I. Bodnarchuk, G. Raino, M. V. Kovalenko, D. J. Norris, R. F. Mahrt, T. Stoferle, Unraveling the origin of the long fluorescence decay component of cesium lead halide perovskite nanocrystals. *ACS Nano* **14**, 14939–14946 (2020).

40. D. Rossi, T. Qiao, X. Liu, M. Khurana, A. V. Akimov, J. Cheon, D. H. Son, Size-dependent dark exciton properties in cesium lead halide perovskite quantum dots. *J. Chem. Phys.* **153**, 184703 (2020).

41. P. Tamarat, E. Prin, Y. Berezovska, A. Moskalenko, T. P. T. Nguyen, C. Xia, L. Hou, J.-B. Trebbia, M. Zacharias, L. Pedesseau, C. Katan, M. I. Bodnarchuk, M. V. Kovalenko, J. Even, B. Lounis, Universal scaling laws for charge-carrier interactions with quantum confinement in lead-halide perovskites. *Nat. Commun.* **14**, 229 (2023).

42. C. R. Kagan, C. B. Murray, M. G. Bawendi, Long-range resonance transfer of electronic excitations in close-packed CdSe quantum-dot solids. *Phys. Rev. B* **54**, 8633–8643 (1996).

43. C. R. Kagan, C. B. Murray, M. Nirmal, M. G. Bawendi, Electronic Energy Transfer in CdSe Quantum Dot Solids. *Phys. Rev. Lett.* **76**, 1517–1520 (1996).

44. S. A. Crooker, J. A. Hollingsworth, S. Tretiak, V. I. Klimov, Spectrally resolved dynamics of energy transfer in quantum-dot assemblies: Towards engineered energy flows in artificial materials. *Phys. Rev. Lett.* **89**, 186802 (2002).

45. J. M. Richter, F. Branchi, F. V. de Almeida Camargo, B. Zhao, R. H. Friend, G. Cerullo, F. Deschler, Ultrafast carrier thermalization in lead iodide perovskite probed with two-dimensional electronic spectroscopy. *Nat. Commun.* **8**, 376 (2017).

46. M. Li, S. Bhaumik, T. W. Goh, M. S. Kumar, N. Yantara, M. Gratzel, S. Mhaisalkar, N. Mathews, T. C. Sum, Slow cooling and highly efficient extraction of hot carriers in colloidal perovskite nanocrystals. *Nat. Commun.* **8**, 14350 (2017).

47. M. S. Kirschner, B. T. Diroll, P. Guo, S. M. Harvey, W. Helweh, N. C. Flanders, A. Brumberg, N. E. Watkins, A. A. Leonard, A. M. Evans, M. R. Wasielewski, W. R. Dichtel, X. Zhang, L. X. Chen, R. D. Schaller, Photoinduced, reversible phase transitions in all-inorganic perovskite nanocrystals. *Nat. Commun.* **10**, 504 (2019).

48. F. Krieg, S. T. Ochsenbein, S. Yakunin, S. Ten Brinck, P. Aellen, A. Suess, B. Clerc, D. Guggisberg, O. Nazarenko, Y. Shynkarenko, S. Kumar, C. J. Shih, I. Infante, M. V. Kovalenko, Colloidal CsPbX3 (X = Cl, Br, I) Nanocrystals 2.0: Zwitterionic capping ligands for improved durability and stability. *ACS Energy Lett.* **3**, 641–646 (2018).

49. A. Loiudice, S. Saris, E. Oveisi, D. T. L. Alexander, R. Buonsanti, CsPbBr3QD/AlOxInorganic nanocomposites with exceptional stability in water, light, and heat. *Angew. Chem. Int. Ed. Engl.* **56**, 10696–10701 (2017).

50. A. Loiudice, S. Saris, R. Buonsanti, Tunable metal oxide shell as a spacer to study energy transfer in semiconductor nanocrystals. *J. Phys. Chem. Lett.* **11**, 3430–3435 (2020).

51. A. L. Efros, M. Rosen, Random telegraph signal in the photoluminescence intensity of a single quantum dot. *Phys. Rev. Lett.* **78**, 1110–1113 (1997).

52. J. A. McGuire, M. Sykora, I. Robel, L. A. Padilha, J. Joo, J. M. Pietryga, V. I. Klimov, Spectroscopic signatures of photocharging due to hot-carrier transfer in solutions of semiconductor nanocrystals under low-intensity ultraviolet excitation. *ACS Nano* **4**, 6087–6097 (2010).

53. F. T. Rabouw, M. Kamp, R. J. van Dijk-Moes, D. R. Gamelin, A. F. Koenderink, A. Meijerink, D. Vanmaekelbergh, Delayed exciton emission and its relation to blinking in Cdse quantum dots. *Nano Lett.* **15**, 7718–25 (2015).

54. K. E. Shulenberger, H. R. Keller, L. M. Pellows, N. L. Brown, G. Dukovic, Photocharging of colloidal CdS nanocrystals. *J. Phys. Chem. C* **125**, 22650–22659 (2021).

55. M. Delor, A. H. Slavney, N. R. Wolf, M. R. Filip, J. B. Neaton, H. I. Karunadasa, N. S. Ginsberg, Carrier diffusion lengths exceeding 1 μm despite trap-limited transport in halide double perovskites. *ACS Energy Lett.* **5**, 1337–1345 (2020).

56. H. Zhu, K. Miyata, Y. Fu, J. Wang, P. P. Joshi, D. Niesner, K. W. Williams, S. Jin, X.-Y. Zhu, Screening in crystalline liquids protects energetic carriers in hybrid perovskites. *Science* **353**, 1409–1413 (2016).

57. X. Y. Zhu, V. Podzorov, Charge carriers in hybrid organic-inorganic lead halide perovskites might be protected as large polarons. *J. Phys. Chem. Lett.* **6**, 4758–61 (2015).

58. C. D. Sonnichsen, D. P. Strandell, P. J. Brosseau, P. Kambhampati, Polaronic quantum confinement in bulk CsPbBr3 perovskite crystals revealed by state-resolved pump/probe spectroscopy. *Phys. Rev. Res.* **3**, 023147 (2021).

59. F. Ambrosio, J. Wiktor, F. De Angelis, A. Pasquarello, Origin of low electron–hole recombination rate in metal halide perovskites. *Energ. Environ. Sci.* **11**, 101–105 (2018).

60. M. J. Schilcher, P. J. Robinson, D. J. Abramovitch, L. Z. Tan, A. M. Rappe, D. R. Reichman, D. A. Egger, The significance of polarons and dynamic disorder in halide perovskites. *ACS Energy Lett.* **6**, 2162–2173 (2021).

61. D. T. Limmer, N. S. Ginsberg, Photoinduced phase separation in the lead halides is a polaronic effect. *J. Chem. Phys.* **152**, 230901 (2020).

62. Q. Qian, Z. Wan, H. Takenaka, J. K. Keum, T. J. Smart, L. Wang, P. Wang, J. Zhou, H. Ren, D. Xu, Y. Huang, Y. Ping, X. Duan, Photocarrier-induced persistent structural polarization in soft-lattice lead halide perovskites. *Nat. Nanotechnol.* **18**, 357–364 (2023).

63. J. N. Wilson, J. M. Frost, S. K. Wallace, A. Walsh, Dielectric and ferroic properties of metal halide perovskites. *APL Mater.* **7**, 010901 (2019).

64. M. C. Brennan, J. E. Herr, T. S. Nguyen-Beck, J. Zinna, S. Draguta, S. Rouvimov, J. Parkhill, M. Kuno, Origin of the size-dependent stokes shift in CsPbBr3 perovskite nanocrystals. *J. Am. Chem. Soc.* **139**, 12201–12208 (2017).

65. W. Shcherbakov-Wu, P. C. Sercel, F. Krieg, M. V. Kovalenko, W. A. Tisdale, Temperature-independent dielectric constant in CsPbBr3 nanocrystals revealed by linear absorption spectroscopy. *J. Phys. Chem. Lett.* **12**, 8088–8095 (2021).

66. F. Krieg, P. C. Sercel, M. Burian, H. Andrusiv, M. I. Bodnarchuk, T. Stöferle, R. F. Mahrt, D. Naumenko, H. Amenitsch, G. Rainò, M. V. Kovalenko, Monodisperse long-chain sulfobetaine-capped CsPbBr3 nanocrystals and their superfluorescent assemblies. *ACS Cent. Sci.* **7**, 135–144 (2020).

67. J. Kang, L. W. Wang, High defect tolerance in lead halide perovskite CsPbBr3. *J. Phys. Chem. Lett.* **8**, 489–493 (2017).

68. Q. A. Akkerman, G. Raino, M. V. Kovalenko, L. Manna, Genesis, challenges and opportunities for colloidal lead halide perovskite nanocrystals. *Nat. Mater.* **17**, 394–405 (2018).

69. M. I. Saidaminov, K. Williams, M. Wei, A. Johnston, R. Quintero-Bermudez, M. Vafaie, J. M. Pina, A. H. Proppe, Y. Hou, G. Walters, S. O. Kelley, W. A. Tisdale, E. H. Sargent, Multi-cation perovskites prevent carrier reflection from grain surfaces. *Nat. Mater.* **19**, 412–418 (2020).

70. Samuel D. Stranks, Giles E. Eperon, Giulia Grancini, Christopher Menelaou, Marcelo J. P. Alcocer, Tomas Leijtens, Laura M. Herz, Annamaria Petrozza, Henry J. Snaith, Electron-hole diffusion lengths exceeding 1 Micrometer in an organometal trihalide perovskite absorber. *Science* **342**, 341–344 (2013).

71. E. M. Y. Lee, W. A. Tisdale, A. P. Willard, Perspective: Nonequilibrium dynamics of localized and delocalized excitons in colloidal quantum dot solids. *J. Vac. Sci. Technol. A* **36**, 068501 (2018).

72. M. K. Gangishetty, S. Hou, Q. Quan, D. N. Congreve, Reducing architecture limitations for efficient blue perovskite light-emitting diodes. *Adv. Mater.* **30**, e1706226 (2018).

73. J. Ye, Z. Li, D. J. Kubicki, Y. Zhang, L. Dai, C. Otero-Martinez, M. A. Reus, R. Arul, K. R. Dudipala, Z. Andaji-Garmaroudi, Y. T. Huang, Z. Li, Z. Chen, P. Muller-Buschbaum, H. L. Yip, S. D. Stranks, C. P. Grey, J. J. Baumberg, N. C. Greenham, L. Polavarapu, A. Rao, R. L. Z. Hoye, Elucidating the role of antisolvents on the surface chemistry and optoelectronic properties of CsPbBrxI3-xPerovskite nanocrystals. *J. Am. Chem. Soc.* **144**, 12102–12115 (2022).

74. Y.-H. Kim, S. Kim, A. Kakekhani, J. Park, J. Park, Y.-H. Lee, H. Xu, S. Nagane, R. B. Wexler, D.-H. Kim, S. H. Jo, L. Martínez-Sarti, P. Tan, A. Sadhanala, G.-S. Park, Y.-W. Kim, B. Hu, H. J. Bolink, S. Yoo, R. H. Friend, A. M. Rappe, T.-W. Lee, Comprehensive defect suppression in perovskite nanocrystals for high-efficiency light-emitting diodes. *Nat. Photonics* **15**, 148–155 (2021).

75. J. H. Park, A. Y. Lee, J. C. Yu, Y. S. Nam, Y. Choi, J. Park, M. H. Song, Surface ligand engineering for efficient perovskite nanocrystal-based light-emitting diodes. *ACS Appl. Mater. Interfaces* **11**, 8428–8435 (2019).

76. F. Yan, J. Xing, G. Xing, L. Quan, S. T. Tan, J. Zhao, R. Su, L. Zhang, S. Chen, Y. Zhao, A. Huan, E. H. Sargent, Q. Xiong, H. V. Demir, Highly efficient visible colloidal lead-halide perovskite nanocrystal light-emitting diodes. *Nano Lett.* **18**, 3157–3164 (2018).

77. L. Wang, B. Liu, X. Zhao, H. V. Demir, H. Gu, H. Sun, Solvent-assisted surface engineering for high-performance all-inorganic perovskite nanocrystal light-emitting diodes. *ACS Appl. Mater. Interfaces* **10**, 19828–19835 (2018).

78. X. Li, Y. Wu, S. Zhang, B. Cai, Y. Gu, J. Song, H. Zeng, CsPbX3Quantum dots for lighting and displays: Room-temperature synthesis, photoluminescence superiorities, underlying origins and white light-emitting diodes. *Adv. Funct. Mater.* **26**, 2435–2445 (2016).

79. J. Pan, L. N. Quan, Y. Zhao, W. Peng, B. Murali, S. P. Sarmah, M. Yuan, L. Sinatra, N. M. Alyami, J. Liu, E. Yassitepe, Z. Yang, O. Voznyy, R. Comin, M. N. Hedhili, O. F. Mohammed, Z. H. Lu, D. H. Kim, E. H. Sargent, O. M. Bakr, Highly efficient perovskite-quantum-dot light-emitting diodes by surface engineering. *Adv. Mater.* **28**, 8718–8725 (2016).

80. Y.-S. Park, J. Roh, B. T. Diroll, R. D. Schaller, V. I. Klimov, Colloidal quantum dot lasers. *Nat. Rev. Mater.* **6**, 382–401 (2021).

81. S. Yakunin, L. Protesescu, F. Krieg, M. I. Bodnarchuk, G. Nedelcu, M. Humer, G. De Luca, M. Fiebig, W. Heiss, M. V. Kovalenko, Low-threshold amplified spontaneous emission and lasing from colloidal nanocrystals of caesium lead halide perovskites. *Nat. Commun.* **6**, 8056 (2015).

82. C.-Y. Huang, C. Zou, C. Mao, K. L. Corp, Y.-C. Yao, Y.-J. Lee, C. W. Schlenker, A. K. Y. Jen, L. Y. Lin, CsPbBr3Perovskite quantum dot vertical cavity lasers with low threshold and high stability. *ACS Photonics* **4**, 2281–2289 (2017).

83. S. Saris, A. Loiudice, M. Mensi, R. Buonsanti, Exploring energy transfer in a metal/perovskite nanocrystal antenna to drive photocatalysis. *J. Phys. Chem. Lett.* **10**, 7797–7803 (2019).

84. A. Loiudice, M. Strach, S. Saris, D. Chernyshov, R. Buonsanti, Universal oxide shell growth enables in situ structural studies of perovskite nanocrystals during the anion exchange reaction. *J. Am. Chem. Soc.* **141**, 8254–8263 (2019).

85. J. Maes, L. Balcaen, E. Drijvers, Q. Zhao, J. De Roo, A. Vantomme, F. Vanhaecke, P. Geiregat, Z. Hens, Light absorption coefficient of cspbbr3perovskite nanocrystals. *J. Phys. Chem. Lett.* **9**, 3093–3097 (2018).

86. Y. Yang, M. Yang, D. T. Moore, Y. Yan, E. M. Miller, K. Zhu, M. C. Beard, Top and bottom surfaces limit carrier lifetime in lead iodide perovskite films. *Nat. Energy* **2**, 16207 (2017).

87. A. J. Mork, M. C. Weidman, F. Prins, W. A. Tisdale, Magnitude of the förster radius in colloidal quantum dot solids. *J. Phys. Chem. C* **118**, 13920–13928 (2014).

88. Z. Yang, A. Surrente, K. Galkowski, A. Miyata, O. Portugall, R. J. Sutton, A. A. Haghighirad, H. J. Snaith, D. K. Maude, P. Plochocka, R. J. Nicholas, Impact of the halide cage on the electronic properties of fully inorganic cesium lead halide perovskites. *ACS Energy Lett.* **2**, 1621–1627 (2017).

89. D. Lapkin, C. Kirsch, J. Hiller, D. Andrienko, D. Assalauova, K. Braun, J. Carnis, Y. Y. Kim, M. Mandal, A. Maier, A. J. Meixner, N. Mukharamova, M. Scheele, F. Schreiber, M. Sprung, J. Wahl, S. Westendorf, I. A. Zaluzhnyy, I. A. Vartanyants, Spatially resolved fluorescence of caesium lead halide perovskite supercrystals reveals quasi-atomic behavior of nanocrystals. *Nat. Commun.* **13**, 892 (2022).
